# Longitudinal multiomics analysis of aggressive pituitary neuroendocrine tumors: comparing primary and recurrent tumors from the same patient, reveals genomic stability and heterogeneous transcriptomic profiles with alterations in metabolic pathways

**DOI:** 10.1186/s40478-024-01796-x

**Published:** 2024-08-31

**Authors:** Keiko Taniguchi-Ponciano, Silvia Hinojosa-Alvarez, Jesus Hernandez-Perez, Rocio A. Chavez-Santoscoy, Ilan Remba-Shapiro, Gerardo Guinto, Erika Magallon-Gayon, Benjamin Telles-Ramirez, Rodrigo Ponce de Leon-Conconi, Sandra Vela-Patiño, Sergio Andonegui-Elguera, Amayrani Cano-Zaragoza, Florencia Martinez-Mendoza, Jacobo Kerbel, Marco Loza-Mejia, Juan Rodrigo-Salazar, Alonso Mendez-Perez, Cristina Aguilar-Flores, Antonieta Chavez-Gonzalez, Elenka Ortiz-Reyes, Erick Gomez-Apo, Laura C. Bonifaz, Daniel Marrero-Rodriguez, Moises Mercado

**Affiliations:** 1 Unidad de Investigación Médica en Enfermedades Endocrinas, Hospital de Especialidades, Centro Médico Nacional Siglo XXI, Instituto Mexicano del Seguro Social, Av. Cuauhtémoc 330, Col. Doctores, Ciudad de Mexico, 06720 México; 2 Escuela de Ingeniería y Ciencias, Tecnológico de Monterrey, Monterrey, México; 3 Centro Neurológico, Centro Médico ABC, Ciudad de Mexico, México; 4 Design, Isolation, and Synthesis of Bioactive Molecules Research Group, Chemical Sciences School, Universidad La Salle-México, Mexico City, Mexico; 5 Unidad de Investigación Médica en Inmunología, Hospital de Pediatría, Centro Médico Nacional Siglo XXI, Instituto Mexicano del Seguro Social, Ciudad de Mexico, México; 6 Unidad de Investigación Médica en Enfermedades Oncológicas, Hospital de Oncología, Centro Médico Nacional Siglo XXI, Instituto Mexicano del Seguro Social, Ciudad de Mexico, México; 7 Área de Neuropatología, Servicio de Anatomía Patológica, Hospital General de México Dr. Eduardo Liceaga, Ciudad de Mexico, México; 8 Coordinación de Investigación en Salud, Centro Médico Nacional Siglo XXI, Instituto Mexicano del Seguro Social, Ciudad de Mexico, México; 9 Unidad de Investigación Médica en Inmunoquímica, Hospital de Especialidades, Centro Médico Nacional Siglo XXI, Instituto Mexicano del Seguro Social, Ciudad de Mexico, México

**Keywords:** PitNET, Recurrent, Aggressive, Transcriptome, Same patient, Exome

## Abstract

Pituitary neuroendocrine tumors (PitNET) represent the vast majority of sellar masses. Some behave aggressively, growing rapidly and invading surrounding tissues, with high rates of recurrence and resistance to therapy. Our aim was to establish patterns of genomic, transcriptomic and methylomic evolution throughout time in primary and recurrent tumors from the same patient. Therefore, we performed transcriptome- and exome-sequencing and methylome microarrays of aggressive, primary, and recurrent PitNET from the same patient. Primary and recurrent tumors showed a similar exome profile, potentially indicating a stable genome over time. In contrast, the transcriptome of primary and recurrent PitNET was dissimilar. Gonadotroph, silent corticotroph, as well as metastatic corticotroph and a somatotroph PitNET expressed genes related to fatty acid biosynthesis and metabolism, phosphatidylinositol signaling, glycerophospholipid and phospholipase D signaling, respectively**.** Diacylglycerol kinase gamma (DGKG), a key enzyme in glycerophospholipid metabolism and phosphatidylinositol signaling pathways, was differentially expressed between primary and recurrent PitNET. These alterations did not seem to be regulated by DNA methylation, but rather by several transcription factors. Molecular docking showed that dasatinib, a small molecule tyrosine kinase inhibitor used in the treatment of chronic lymphocytic and acute lymphoblastic leukemia, could target DGKG. Dasatinib induced apoptosis and decreased proliferation in GH3 cells. Our data indicate that pituitary tumorigenesis could be driven by transcriptomically heterogeneous clones, and we describe alternative pharmacological therapies for aggressive and recurrent PitNET.

**Supplementary Information:**

The online version contains supplementary material available at 10.1186/s40478-024-01796-x.

## Background

Pituitary neuroendocrine tumors (PitNET) represent 15% of all intracranial tumors. These neoplasms can be classified as clinically functioning and non-functioning PitNET [1, 2]. Clinically functioning tumors result in specific hormonal hypersecretion syndromes, according to their cell of origin: acromegaly/gigantism due to somatotroph PitNET; hyperprolactinemia due to lactotroph PitNET, which in females causes amenorrhea/galactorrhea and in males sexual dysfunction; Cushing disease due to corticotroph PitNET, and central hyperthyroidism due to the rare thyrotroph PitNET [1, 2]. Over 60% of non-functioning PitNET are of gonadotroph differentiation and immunostain for α-subunit, LH-β and/or FSH-β, although seldom do they give rise to a clinically distinct hormonal hypersecretion syndrome [3]. Some PitNET exhibit an aggressive behavior, growing rapidly and invading surrounding tissues, and are frequently resistant to multimodal treatment [4]. PitNET often recur after initial surgery, particularly when invasive, precluding complete resection of the lesion, which can only be achieved in ~ 40–50% of all patients [5, 6]. More than 10–20% of cases with gross tumor resection will experience a relapse 5 to 10 years after the operation. This number rises to 40% and 50% at 5 years and 10 years, respectively if there is residual tumor after the initial operation. Overall recurrence rates are 25%, 44% and 64%, at 5, 10 and 15 years, respectively [5, 6]. There are currently no reliable markers to predict recurrence.

The molecular and cellular pathogenesis of PitNET recurrence is still largely unknown. In the present work, we carried out comprehensive whole exome and transcriptome sequencing analysis as well as methylation profiling of paired primary and recurrent PitNET from the same patient to identify molecular markers of recurrence-persistence, seeking patterns of genome evolution, as well as transcriptomic and methylomic changes over time.

## Methods

### Patients and tissue samples

#### Primary and recurrent cohort from the same patient

A total of 11 patients with paired primary and recurrent-persistent PitNET were included in the study: five females and three males with non-functioning, gonadotroph PitNET; a female patient with a silent corticotroph PitNET, a female with acromegaly due to a somatotroph PitNET, and another female patient with Cushing disease due to a metastatic corticotroph PitNET. All tumors included in the study were sporadic and were collected from patients diagnosed, treated, and followed at the Endocrinology Service and the Neurosurgical department of Hospital de Especialidades, Centro Médico Nacional Siglo XXI of the Instituto Mexicano del Seguro Social. The study protocol was approved by the Comisión Nacional de Ética e Investigación Científica del Instituto Mexicano del Seguro Social (approval: R-2022–3601-186 and R-2019–785-052) and it was caried out in accordance with the Helsinki declaration principles. All participating patients signed an informed consent.

#### Immunophenotyping of PitNET: Immunohistochemistry for hormones and transcription factors (TF)

The World Health Organization 2022 diagnostic guidelines were used to classify the tumors. Paraffin-embedded, formalin-fixed tissue blocks were obtained and 3 μm sections were stained with hematoxylin–eosin and reviewed by a neuro-pathologist. Tumors were represented with a twofold redundancy. Sections were cut and placed onto coated slides. Immunostaining was performed by means of the HiDef detection HRP polymer system (Cell Marque, CA, USA), using specific antibodies against each pituitary hormone (TSH, GH, PRL, FSH, LH and ACTH) and the lineage specific TFs TBX19 (T-PIT), POU1F1 (PIT-1) and NR5A1 (SF1), as previously described [7]. Interpretation of immunohistochemistry for pituitary hormones and TF was carried out by two independent observers.

#### Immunofluorescence assays and confocal microscopy

Paraffin-embedded, formalin-fixed tissue blocks were stained with hematoxylin–eosin and reviewed by a pathologist. Sections (3 μm) were cut and placed onto coated slides. Remaining paraffin in the slides was removed at 70ºC for 40 min. A train of solvents (Xylol/Ethanol) was used for rehydration of the tissues. Heat-induced antigen retrieval was performed (citrate buffer pH 6.0, sodium citrate 10 μM) at 120ºC for 20 min. Tissue was permeabilized for 2 h (10 mg/mL bovine serum albumin, 5% horse serum, 0.02% sodium azide, and 0.5% Triton). Permeabilized tissue was incubated for 18 h with primary anti DGKG antibody (ab151967, Abcam) and TUBB3 (ab231083, Abcam). Incubation of secondary anti-rabbit 488 and anti-mouse AF594 antibodies (Jackson Immunoresearch) was performed for 2 h. Nuclei were stained with Hoechst 33,342 reagent or DAPI (4′,6-diamidino-2-phenylindole) (Invitrogen) for 10 min. Finally, tissues were mounted with Vectashield (Vector Laboratories). Images were obtained on a Nikon Ti Eclipse inverted confocal microscope (Nikon Corporation) using NIS Elements v.4.50. Imaging was carried out using a 20x (dry, NA 0.8) objective lens. Zoom was performed at 3.4x. Images were analyzed using the FIJI ImageJ Software.

#### DNA purification

Pituitary tissue was lysed in proteinase K solution. After lysis, 300 μL of 5 M ammonium acetate was added to precipitate proteins and cellular components. The aqueous phase was transferred to a fresh tube, 600 μL of isopropanol were added and the mixture was incubated overnight at -20 °C. The mixture was then centrifuged at 14 000 rpm for 30 min. The resulting DNA pellet was washed with 1 mL 75% ethanol and centrifuged at 10 000 rpm for 5 min; the pellet was air-dried, and the DNA resuspended in nuclease free water [7].

#### RNA purification

Total RNA was extracted from pituitary tissue using the miRNAeasy Mini Kit (Qiagen Inc, CA, USA) according to manufacturer’s instructions. Tissue samples were disrupted and homogenized in 700 μL Qiazol Lysis Reagent. They were then incubated at room temperature for 5 min. Next, 200 μL of chloroform were added, and samples were incubated at room temperature for 3 min. The mixture was centrifuged at 12 500 rpm for 15 min at 4 °C. The aqueous phase was transferred to a fresh tube and mixed with an equal volume of 70% ethanol. Samples were then transferred to an RNAeasy Column in a 2 mL tube and centrifuged at 10 000 rpm for 15 s. After centrifugation, 700 μL of RW1 buffer were added and the mixture was centrifuged at 10 000 rpm for 15 s. Flow-through was discarded and 500 μL of RPE buffer was added to the membrane and then centrifuged at 10 000 rpm for 15 s (2x). The column was transferred to a new collection tube adding 30 μL of RNAse free water and centrifuged for 1 min at 10 000 rpm. RNA was quantified using a Nanodrop-ND-1000 spectrophotometer (Thermo Scientific, DE, USA); RNA integrity was evaluated by Bioanalyzer 2100 [7].

#### Whole RNA sequencing

RNA integrity of each sample was assessed using a R1 RNA Cartridge for QSep 400 (BiOptic, New Taipei City, Taiwan), RNA concentration was measured with Qubit RNA HS Assay Kit (Invitrogen, Carlsbad, CA, United States) and purity was analyzed with a NanoDrop 1000 spectrophotometer (Thermo Fisher Scientific, Wilmington, DE). Transcriptome libraries were prepared with the TruSeq Stranded Total RNA Library Prep with Ribo-Zero Gold (Illumina, San Diego CA, United States). Fragmentation times were adjusted based on RIN. Transcriptome libraries were quantified with Qubit dsDNA HS Assay Kit (Invitrogen, Carlsbad, CA, United States), library size was analyzed in S2 Standard DNA Cartridge for QSep 400 (BiOptic, New Taipei City, Taiwan), and sequencing was performed in a NovaSeq 6000 (Illumina, San Diego CA, United States) in a 150 bp pair-end configuration.

#### Whole exome sequencing (WES)

The genomic DNA (gDNA) was shipped to the Genomics Core Lab of the Tecnológico de Monterrey for exome sequencing. gDNA was quantified using Qubit dsDNA BR Assay Kit (Invitrogen, Carlsbad, CA, USA). Quality was determined spectrophotometrically using a Nanodrop One spectrophotometer (Thermo Fisher Scientific, Waltham MA, USA). WES libraries were prepared using Illumina DNA Prep with Exome 1.0 Enrichment (Illumina, San Diego CA, United States). All libraries were quantified with the Qubit dsDNA BR Assay Kit (Invitrogen, Carlsbad, CA, USA), library size was analyzed in S2 Standard DNA Cartridge for Sep 400 (BiOptic, New Taipei City, Taiwan), and sequencing was performed in a NovaSeq 6000 (Illumina, San Diego CA, United States) in a 150 bp pair-end configuration.

#### DNA methylation

The methylation profiles of PitNET were determined by means of the Infinium MethylationEPIC BeadChip Array (Illumina Inc.) following the manufacturer's protocol. Sodium bisulfite modification was performed on 1 μg of DNA using the Zymo EZ DNA methylation kit (Zymo Research). The treated DNA was whole genome amplified and enzymatically fragmented. Finally, the amplified and fragmented DNA was hybridized to the MethylationEPIC BeadChip. The chips were scanned with the Illumina iScan.

#### FastQC and preprocessing

Quality assessment of the RNAseq and exome libraries was performed with FastQC (Babraham Bioinformatics) to determine the quality of the sequencing. All raw sequences passed the initial quality filter. Adapters were removed and a quality and length filter were performed with Trimmomatic 0.40.

#### Differential expression

Preprocessed reads were aligned using STAR against the human reference sequence (hg38). Once the BAM files were obtained, HT-Seq package was used to estimate gene expression whereby a count table is obtained that can be used to perform differential expression analysis. Statistical analysis of differential gene expression (DGE) among the groups was performed using the DESeq2 R package, version 2.13 (R Foundation for Statistical Computing, Vienna, Austria). The false discovery rate was set at (FDR) < 0.01 and a threshold normalized absolute log twofold change > 1.0.

For one-to-one comparisons the NOISeq method was used as it is designed to compute differential expression of RNA-Seq data even when there are no replicates available. To exclude genes with low counts across libraries CPM filter was set to 1 and the chosen normalization method was TPM (Transcripts Per Kilobase Million), using weighted trimmed mean of M-values.

#### Altered pathways identification

To analyze pathway alterations, the PDS (pathway deregulation score) of each path noted in KEGG was calculated using the Pathifier algorithm. Pathifier calculates a PDS for each path for each sample. In each path a n-dimensional space is constructed (n = number of genes in the path), where a main curve that captures the variation of a cloud of points is calculated by non-linear regression, with each point representing each sample and its values of expression of the n genes of the pathway. PDS is the distance of the projection to the main curve of each sample with respect to the projection of normal samples. The analysis of this section was performed using R version 4.2.1. KEGG annotated tracks were downloaded using the getGenesets using the EnrichmentBrowser package. An expression matrix was constructed using expression values ​​and PDS ​​were calculated using the "pathifier" package with the default parameters. The top 25 altered pathways were plotted.

#### Cytoscape iRegulon

Regulon analysis was carried out using the cytoscape app iRegulon. Genes participating in altered metabolic pathways identified by the pathifier analysis were used as input for TF gene regulatory network discovery. Default parameters were used, Homo sapiens database, 10 K motif collection, 1120 ChIP-seq collection, 20 Kb centered around TSS putative regulatory region, maximum FDR on motif similarity 0.001 among others.

#### Computational analysis WES

Preprocessed sequences were aligned to the human reference sequence (hg38) using the Illumina-Dragen Enrichment pipeline (llumina, San Diego CA, United States). This pipeline was set to produce copy number variants (–enable-cnv true). The BAM files resulting from the enrichment were removed from PCR duplicates using Picard Tools (http://broadinstitute.github.io/picard). Each BAM file was used to obtain the somatic variants using the GATK pipeline, and variants where annotated using ANNOVAR according to the following databases: Clinvar, gnomAD, refGene, cytoBand, exac03, avsnp147, dbnsfp30a. The somatic variants were then transformed to MAF using Funkotator from GATK. Additionally, converted annotated variant files were analyzed with Maftools package from R programming language to visualize the landscape of critical mutations.

The mutation analysis was carried out using maftools, from this package the merge_mafs tools were used to combine samples, the mutations were filtered using subsetMaf with the parameter “Variant_Classification =  = Missense_Mutations”. Graphs of mutations in genes of interest were constructed using lollipopPlot to observe mutations in general and lollipopPlot2 to compare mutations by study subgroup. The rainfallPlot tool was used to produce the mutation density plots per chromosome. Subsequently, to compare the number of mutations per analysis group, the annotated mutations matrix extracted from the object produced with merge_mafs was used. Venn diagrams were constructed to compare primary and recurrent-persistent tumors, both by individual sample and by subgroup. Finally, to determine the number of shared mutations, the UpSetR package was used, the object with the mutations extracted in the previous step was used to build these graphs.

#### DNA methylation profiling

Quality control, data normalization and statistical analysis of EPIC arrays (IDAT files) were performed using ShinyÉPICo, a graphical pipeline which is available as an R package at the Bioconductor (http://bioconductor.org/packages/shinyepico) and GitHub (https://github.com/omorante/shinyepico) sites. Bisulfite conversion was used as a control probe test and included in ShinyÉpico to determine whether the conversion rate was above the quality threshold of 2, established by Illumina. The selected normalization method was Quantile and CpH and SNP loci were removed from the analysis. Differentially methylated positions (DMP) and regions (DMR) were determined for each contrast (Functioning and No Functioning) considering recurrence as a covariable. The statistics for each CpG were generated and filtered when a p-value < and an FDR < 0.05 were found. Finally, heatmaps of DMP and DMR were generated using the difference of beta values between the groups in each contrast. Additionally, the methylation analysis was carried out using recurrence as the variable of interest and donor as the covariable.

#### Second independent validation cohort for DGKG expression

DGKG expression was validated in an independent cohort, comprising 42 PitNET, including 20 clinically non-functioning PitNET (14 gonadotroph, 3 null cell and 3 silent corticotroph), 10 somatotroph, 6 corticotroph, 4 thyrotroph, and two lactotroph PitNET. All tissue samples were from treatment naïve patients who had not received radiation therapy or any pharmacological intervention prior to surgery, except from lactotroph PitNET who received the standard treatment with the dopamine agonist cabergoline. In these samples, transcriptome analysis was carried out using microarrays as previously described [7]. Six non-tumoral pituitary glands were obtained from autopsies performed at the Pathology Department of Hospital General de México within 10 h of death and were used as controls.

#### Reverse transcription and qPCR

After purification, 1 μg of total RNA was retro transcribed in a 20 μL final volume reaction with the SuperScript VILO Master Mix (Applied Biosystems, CA, USA), 4 μL of Master Mix were added, and the reaction mixture was incubated at 25 °C for 10 min, 42 °C for 60 min, and 85 °C for 5 min, according to manufacturer protocols. For RT-qPCR of DGKG (Hs00176315_m1), all reagents were purchased from Applied Biosystems (CA, USA), and conditions were as follows: 10 μL of Taqman Universal Master Mix II, 1 μL of each Taqman probe, 200 ng of cDNA in a 20 μL final volume, according to manufacturer’s recommendation. RPLP0 (Hs99999902_m1) was used as endogenous control and all reactions were done in triplicate in the Step one thermal cycler (Applied Biosystems). 2-ΔΔCt relative expression was calculated.

#### Methylation specific PCR

DNA from tumors were sodium bisulfite treated using EZ DNA Methylation Lightning Kit (Zymo research). According to manufacturer’s protocol, 1 µg of DNA was mixed with 130 μL of lightning conversion reagent and incubated at 98 °C for 8 min, 54 °C for 60 min, and 4 °C indefinitely. After incubation, 600 µL of M-binding buffer was added and loaded into the column and centrifugated at 10 000 g for 30 s, the column was washed with M-wash buffer and 200 μL of L-desulphonation buffer was added, and the column was washed with M-wash buffer. Bisulfite-treated DNA was collected in 10 μL of M-elution buffer and used in PCR. Primer FM 5'-GTTTTGCGTTCGGGGGTAGGGTTTC-3' and primer RM 5'-TCTATCTCCGTAACCCGCTACTACGA-3' were used for methylated regions and primer FU 5'-GGTTTTGTGTTTGGGGGTAGGGTTTT-3' and Primer RU 5'-CTATCTATCTCCATAACCCACTACTACA-3' were used for unmethylated regions [8]. PCR was performed using 10 μL of GoTaq Green PCR Master Mix, 20 nmol of each primer and 4 μL of bisulfite-treated DNA in a total volume of 20 μL using the program 40 cycles of 95 °C for 30 s, 60 °C for 30 s, and 72 °C for 30 s.

#### Third validation cohort for DGKG expression

The processed transcriptome data from RNAseq experiments of 134 PitNET from all lineages were downloaded from the MTAB-7768 project from the ArrayExpress webpage (https://www.ebi.ac.uk/biostudies/arrayexpress) and was used to evaluate DGKG gene expression. The dataset is comprised by 8 silent corticotroph, 29 gonadotroph, 8 null cell, 8 silent POU1F1 (PIT-1), 27 somatotroph, 27 corticotroph, 16 lactotroph, 6 thyrotroph and 5 mixed POU1F1 (PIT-1)-tumors.

#### scRNAseq cell populations analysis

Data from single-cell GSE208108 were downloaded from GEO, the downloaded files were originally preprocessed using Cell Ranger software under the GRCh38 reference genome. The single-cell data was analyzed using R packages Seurat, clusterProfiler, and enrichplot. The data were separately analyzed for GSM6337436 and GSM6337438, while GSM6337432 and GSM6337434 were merged using the merge () function. Each Seurat object underwent quality control, whereby cells with fewer than 15 features, less than 500 RNA nCounts, and a mitochondrial percentage greater than 10% were removed. Normalization was performed using SCTransform () with adjustment associated with mitochondrial percentage. Subsequently, dimensionality reduction was carried out using PCA and UMAP for up to 50 dimensions. Clustering was tested with 10, 15, 30, and 50 dimensions to ensure consistent results. Clustering was performed with FindNeighbors and FindClusters using 20 dimensions and a resolution of 0.5. Manual curation of cell group identification was performed using feature plots. In each sample, a Seurat object was constructed containing only the tumor cells from the original objects using the subset () function from the Seurat package. In these new objects, the k-nearest neighbor (KNN) graph was recalculated with edge weights refined by Jaccard similarity using FindNeighbors (). Subsequently, clustering was also calculated using FindClusters (), which is a modularity function in the Seurat package. The new clusters were named "Tumor Cells + cluster number", and for each of them, differentially expressed genes were calculated using FindAllMarkers () with default parameters. The differentially expressed genes then underwent an enrichment algorithm using the clusterProfiler and enrichplot packages based on KEGG pathways, with a p-value threshold of < 0.05. The new cluster labels for tumor cells were integrated into the original Seurat object for visualization purposes.

#### Repurposing new drugs in silico evaluation, molecular docking

A) *Ligands*: The E-Drug database was downloaded and curated. After filtering, 1773 compounds were optimized and pre-hydrogenized using the MMFF94 force field using MolConvert 20.19.2, 2021, ChemAxon (http://www.chemaxon.com) program and saved in *.sdf format.

b) *Target*: Human DGKG tridimensional model was downloaded from AlphaFold (id code: P49619) and was evaluated in SAVES v6.0, recording an Overall Quality Factor of 96.2382.

c) *Virtual Screening*: A report from Aulakh S. et al. suggested that the active site of DGKG is delimited by the following residues: Gly 444, Gly 494, Thr 495, Thr 521, and Arg 599; therefore, the searching site was centered in these residues. Autodock Vina and Molegro Virtual Docker (MVD) were used for virtual screening following the standard procedure suggested by the manufacturer. Briefly, for Autodock Vina, the searching area was a square prism built with a = 22.1 Å and h = 28.7 Å, the Kollman charges for the protein were defined using Autodock Tools, the configuration parameters for exhaustiveness, energy range, and the maximum number of binding modes were the established values given by the program. For MVD, the search area was a sphere with a radius of 15 Å of a sphere. The protonation states, and the assignment of charges on proteins and ligands based on neutral pH were assigned in the standard way with MVD. The default search parameters available in the program were used (MolDock Optimizer algorithm). The theoretical binding affinity was predicted through the Rerank Score for MVD and Kcal/mol for Autodock Vina. PyMol v2.5.2 program and the Protein–Ligand Interaction Profiler (PLIP) web tool was used for graphical representation, visualization of molecular interactions, and electrostatic surface mapping.

The in silico ADMET profiles were generated in ADMETLab 2.0 using the list of SMILES of the curated molecular database. Bearing that the molecules screened were FDA-approved drugs, the primary criterion was whether the molecules could penetrate the Blood Brain Barrier (BBB).

#### Cell culture, tyrosine kinase inhibitors treatment, apoptosis and proliferation assays

GH3 cell line was purchased from the ATCC. All reagents used for cell culture were purchased from Gibco (Foster City, USA) unless otherwise stated.

The GH3 cell line was cultured in 50% of F-12 Dulbecco’s Modified Eagle Medium (DMEM) and 50% of High Glucose DMEM supplemented with 2.5% fetal bovine serum (FBS) and 15% Horse serum and 1% penicillin/streptomycin at 37 °C in 5% CO_2_. K562 cell line was cultured in RPMI supplemented with 10% FBS and 1% penicillin/streptomycin at 37 °C in 5% CO_2_.

To evaluate the effect of imatinib, nilotinib and dasatinib on cell apoptosis, cells were seeded in triplicate in 96-well plate at a density of 100,000 cells (GH3 PitNET cell line and K562 cell line as positive control to tyrosine kinase inhibitor (TKI) treatments). Twenty-four hours after planting, imatinib (2.5, 5, 10 y 15 μM), nilotinib (5, 10 y 15 μM) and dasatinib (1, 2.5 y 5 μM) were added, DMSO was used as vehicle. After 48 h, cells were collected to evaluate the percentage of apoptotic cells using Anexin V-FITC and DAPI (4',6-diamidino-2-phenylindole) by flow cytometer (BD FACS VerseTM).

Proliferation assays were conducted according to the manufacturer instructions using Click-iT EdU Cell proliferation Kit Alexa Fluor 488 (Invitrogen). Briefly, 10,000 cells were seeded in 96-well TC plates (Corning) for flow cytometry evaluation, with RPMI/ DMEM F12-10% FBS. Tyrosine kinase inhibitors (TKIs) were added in different concentrations: imatinib, nilotinib (5 μM, 10μ and 15 μM) and dasatinib (1 μM, 2.5μ and 5 μM). Click-iT EdU coupled with Alexa Fluor 488 fluorochrome was added (50 μM) (Invitrogen) to cell culture with TKI treatments and control. After 48 h of cells were processed for flow cytometry evaluation. Acquisitions were made on a spectral flow cytometer Aurora (Cytek Biosciences).

Data were analyzed using FlowJo software V.10.6.2 and statistical analyses were performed using GraphPad Prism software V.9.0.0.

All treatments were performed in triplicate a minimum of three independent times.

## Results

### Clinical, hormonal and imaging characteristics of patients

The total cohort consisted of 11 patients with paired primary and recurrent-persistent PitNET and included 8 patients (5 females and 3 males) who harbored clinically non-functioning gonadotroph PitNET, one female patient with acromegaly due to a somatotroph PitNET, one female with a silent corticotroph PitNET and yet another female with Cushing disease due to a metastatic corticotroph PitNET. Age at diagnosis varied between 16 and 66 years. All patients in the cohort, except the patient with acromegaly harbored large primary and recurrent lesions that extended cephalically and compressed the optic chiasm and invaded one or both cavernous sinuses. The time elapsed between the diagnosis of the primary and the recurrent tumors varied between 8 and 94 months. It is worth clarifying that in all the patients included in the study, the secondary lesion was both persistent (inoperable cavernous sinus remnants) and recurrent (regrowth of the intrasellar component), except the somatotroph PitNET which did not show any invasion in the primary lesion. Two patients harboring gonadotroph PitNET received radiotherapy before recurrence, and another one was receiving levothyroxine replacement. The patient with acromegaly received somatostatin analog treatment before tumor recurrence. Finally, the patient with the metastatic corticotroph PitNET received numerous treatments throughout her course, including temozolomide, ketoconazole and cabergoline, as well as gamma knife radiosurgery for a prepontine metastasis; unfortunately, none of these succeeded in controlling her multiple recurrences (Table 1).

**Table 1:** Clinical information from the primary and recurrent paired tumors

SAMPLE ID 1	SAMPLE ID 2	Gender	Age at diagnosis	Tumor Type	Cavernous Sinus Invation at 1 and 2 surgeries	Max Diameter (1st)	1st Volume (cm3)	1st PO Remanent	Treatment between tumors	Max Diameter (2nd)	2nd Volume	2nd PO Remanent	2nd Max Diameter (remanent)	Time between surgeries
NF-2006–006	NF-2009–012	Male	25	Gon	(+ / +)	35	42.87	YES	XRT	35	35.7	YES	27	1 year
NF-2007–020	NF-2010–012	Male	42	Gon	(+ / +)	23	9.89	YES	XRT	34	19.04	YES	24	3 years
NF-2007–029	NF-2009–019	Female	46	Gon	(+ / +)	30	15.87	YES	NO	23	9.2	YES	UNKNOWN	2 years
NF-2009–011	NF-2012–034	Female	63	Gon	(+ / +)	29	17.34	YES	NO	13	1.01	NO	7	1 year
NF-2009–026	NF-2010–056	Female	31	Gon	(+ / +)	27	16	YES	XRT	20	3.6	YES	21	1 year
AC-2010–010	AC-2011–005	Female	56	Soma	(-/ +)	NO MRI	-	NO MRI	NO	No MRI	-	YES	33	1 year
NF-2013–006	NF-2015–007	Male	20	Gon	(+ / +)	30	22.5	YES	NO	20	7.2	NO	14	2 years
CU-2013–005	CP-2021–001	Female	29	Met corth	(+ / +)	20	4.5	YES	XRT, TMZ, KETO, CABERG	18	4.6	YES	20	8 years
NF-2016–032	NF-2022–013	Female	39	Gon	(+ / +)	36	16.38	YES	NO	28	34.4	YES	35	5 years
NF-2018–018	NF-2022–046	Female	50	Sil corth	(+ / +)	33	24.94	YES	NO	36	37.9	YES	26	4 years
NF-2019–033	NF-2021–024	Female	66	Gon	(+ / +)	23	7.63	YES	LEVO	27	8.72	YES	27	2 years

*Tumor Type: Gon* gonadotroph, *Soma* somatotroph, *Met Corth* metastatic corticotroph, *Sil corth* silent corticotroph

*PO* post operative, *XRT* radiotherapy, *TMZ* temozolomide, *KETO* ketoconazole, *CABERG* cabergoline, *LEVO* levothyroxine

### Primary and recurrent tumors diverge at the transcriptional level

Our first aim was to establish the transcriptional relationship between primary and recurrent tumor from the same patient, performing RNAseq analysis. The primary and recurrent tumors were deep sequenced between 45 and 100 million reads per sample, with approximately 95% correctly mapped reads coding and non-coding. Comparing primary with recurrent tumors, a variable number of differentially expressed genes (DEG) were found among the different tumor types: 3600 in non-functioning PitNET of gonadotroph differentiation, 2570 in the metastatic corticotroph PitNET, 3011 in the silent corticotroph PitNET, and 4400 in the somatotroph PitNET.

**Fig. 1:**
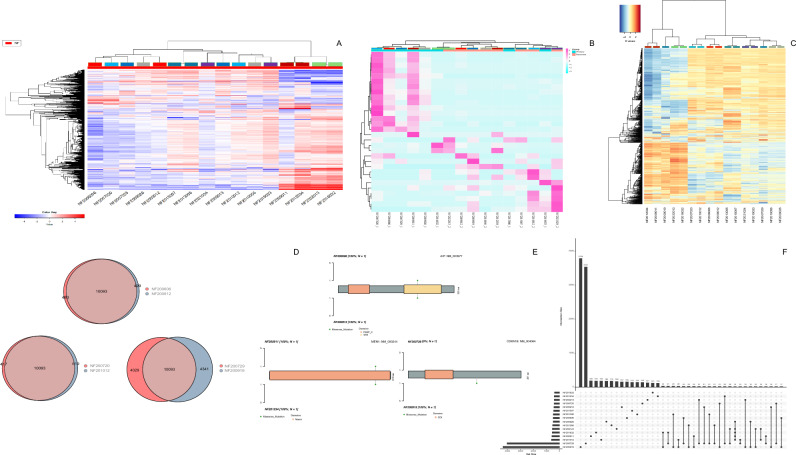
Panel **A** heatmap of differentially expressed genes, panel **B** differentially expressed lincRNA, and panel **C** differentially methylated regions between primary and recurrent gonadotroph PitNET. These tumors correspond to the first cohort whereby primary and recurrent tumors come from the same patient. Each colored rectangle in the upper section of the heatmap denotes the patient, the lower section of the heatmap shows the code: NF = Non-functioning followed by the year and internal control number (e.g. NF2009011 and NF2012034 are primary and recurrent tumors from the same patient). Panels **D**, and **F** Venn diagrams and upset R graphics representing subtle single nucleotide variants differences between primary and recurrent gonadotroph PitNET. Panel **E** lollipop graphics representing missense single nucleotide variants translated at the protein level showing no differences between primary and recurrent tumors. Only one gonadotroph PitNET. showed a high degree of heterogeneity

**Fig. 2:**
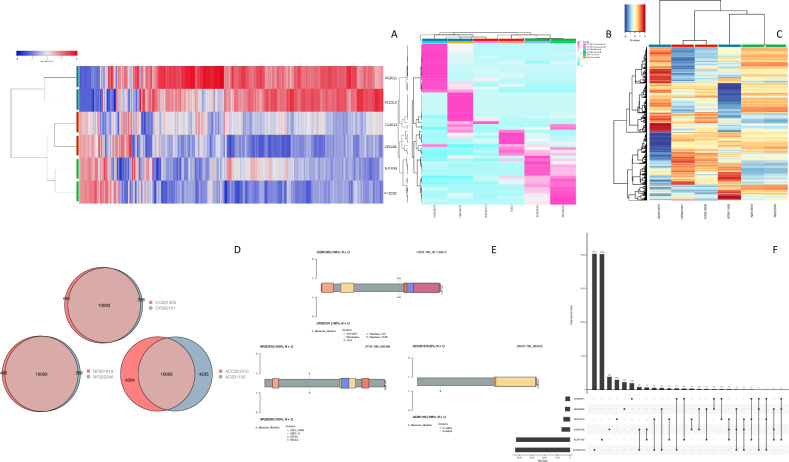
Panel **A** heatmap of differentially expressed genes, panel **B** heatmap of differentially expressed lincRNA, and panel **C** heatmap of differentially methylated regions between primary and recurrent tumors from metastatic corticotroph, somatotroph and silent corticotroph PitNET. These tumors correspond to the first cohort of primary and recurrent tumors from the same patient. Each colored rectangle in the upper section of the heatmap denotes the patient, lower section of the heatmap shows the code: NF = Non-functioning silent corticotroph, PitNET CU = primary metastatic corticotroph PitNET and CP = recurrent metastatic corticotroph PitNET, AC = somatotroph PitNET, followed by the year and internal control number (e.g. NF2018 and NF2022 are primary and recurrent tumors from the same patient). Panels **D**, and **F** Venn diagrams and upset R graphics representing subtle single nucleotide variant differences between primary and recurrent tumors from the metastatic corticotroph PitNET patient, as well as the somatotroph PitNET and the silent corticotroph PitNET patients. Panel **E** lollipop graphics representing missense single nucleotide variants translated at protein level showing no differences between primary and recurrent tumors. Somatotroph PitNET showed a high degree of heterogeneity at SNV

We and others have recently shown that transcriptomically, PitNET cluster according to the TF that determines their terminal differentiation as NR5A1 (SF1)-derived gonadotroph tumors, TBX19 (T-PIT)-driven ACTH-tumors and POU1F1 (PIT-1)-derived GH-, PRL- and TSH-secreting tumors [7] (Additional file 1: Fig. S1). Thus, we analyzed tumors derived from each of these lineages separately. Variable degrees of transcriptomic heterogeneity were found among patients with different tumor types and between primary and recurrent lesions. Interestingly, such heterogeneity was independent of the TF driving each tumor type (Figs. 1A, 2A) (Additional file 1: Fig. S1).

**Fig. 3:**
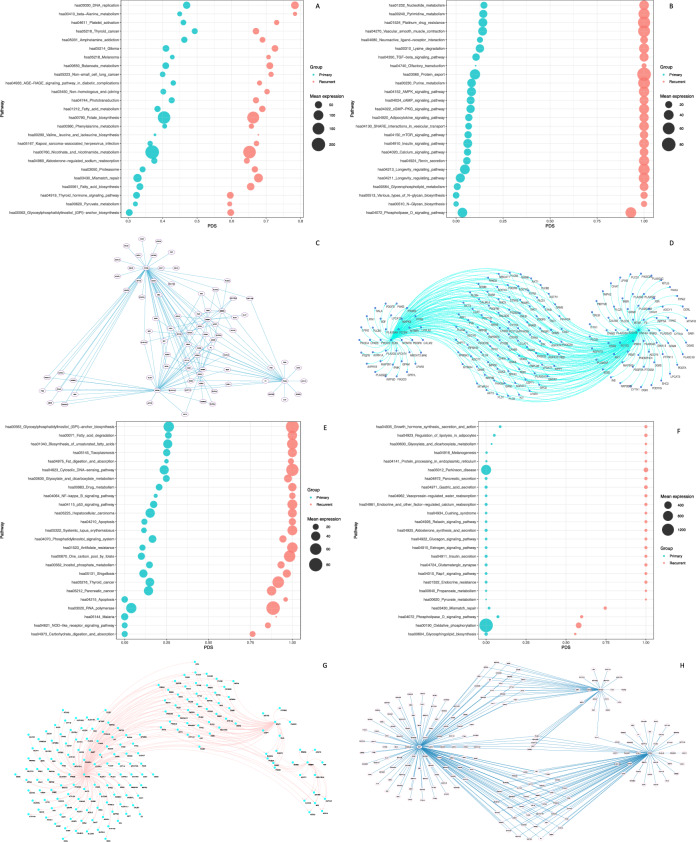
Panels **A** and **C** bubble plot of altered metabolic pathways in gonadotroph PitNET showing how the transcription factors PPARG, ZBTB3 and SREBF1-2 can potentially regulate the expression of metabolic genes expressed in these pituitary neuroendocrine tumors. Panel **B** bubble plot of dysregulated pathways in metastatic corticotroph PitNET, and panel **D** gene regulatory networks in metastatic corticotroph PitNET Panel **E** bubble plot of dysregulated pathways in somatotroph PitNET and panel **G** gene regulatory network of somatotroph PitNET. Panel **F** bubble plot of dysregulated pathways in silent corticotroph PitNET and panel **H** gene regulatory network of silent corticotroph PitNET

Most of the patients included in the study harbored clinically non-functioning PitNET of gonadotroph differentiation. Transcriptomic analysis revealed significant differences between primary and recurrent tumors. Most of primary tumors clustered together and separate from the recurrent tumors which also clustered together among themselves in most cases. Interestingly in the recurrent tumors, altered genes are related to metabolic pathways such as fatty acid biosynthesis (*ACACA, p* = *0.002*) and metabolism (*ELOVL7, p* = *0.03*), pyruvate (*LDHC, p* = *0.020*), and phenylalanine metabolism (*ALDH3B2, p* = *0.031*), as well as valine, leucine and isoleucine biosynthesis (*BCAT1, p* = *0.004*) (Fig. 1). Tumor related events such as DNA replication (*EXO1, p* = *0.036*) and mismatch repair (*POLD3, p* = *0.011*) were altered in primary and recurrent tumors (Fig. 3A).

As we have previously shown [7], the gene expression profile of the metastatic corticotroph PitNET and the silent corticotroph PitNET differed significantly (Fig. 2A). The metastatic corticotroph PitNET showed alterations in molecules involved in various types of N-glycan biosynthesis (*MAN1A2, p* = *0.014*), glycerophospholipid metabolism (*DGKG, p* = *0.015*), phospholipase D signaling (*DGKG, p* = *0.015*), purine (*PDE1C, p* = *0.000*), pyrimidine (*DPYD, p* = *0.036*), and nucleotide metabolism (*DGUOK, p* = *0.013*) (Fig. 3B). In contrast, the silent corticotroph PitNET showed abnormalities in the expression of genes involved in phosphatidylinositol signaling (*DGKG, p* = *0.015*), inositol phosphate metabolism (*INPP5A, p* = *0.027*), carbohydrate digestion and absorption (*HK2, p* = *0.05*), fatty acid degradation (*ADH5, p* = *0.047*) and biosynthesis of unsaturated fatty acids (*SCD, p* = *0.03*) (Fig. 3F). The somatotroph PitNET showed an altered expression of genes involved in different pathways, including pyruvate metabolism (*PCK1, p* = *0.013*), phospholipase D signaling (DGKG, *p* = *0.023*), propanoate metabolism (*ACSS3, p* = *0.04*), insulin secretion (*ATP1A3, p* = *0.003*), aldosterone synthesis and secretion (*CAMK1D, p* = *0.009*), endocrine and other factor-regulated calcium reabsorption (*ESR1, p* = *0.033*), as well as protein processing in the endoplasmic reticulum (*EDEM3, p* = *0.03*) (Fig. 3E).

In addition to mutations or aberrant expression in protein coding genes, the dysregulation of long non-coding RNA (LINC) appears to play a role in tumorigenesis [9]. Therefore, we analyzed the expression of long non-coding RNA (lincRNA) from tumors whole transcriptome to search for expression patterns in recurrent tumors. Primary and recurrent tumors had different lincRNA expression profiles. Each recurrent tumor showed upregulation of a distinct lincRNA: *LINC01619 (p* = *0.014)* in the metastatic corticotroph PitNET, *LINC00342 (p* = *0.037)* in the silent corticotroph PitNET, *LINC02691 (p* = *0.000)* in the somatotroph PitNET, and *LINC00486 (p* = *0.0006)* in the clinically nonfunctioning PitNET of gonadotroph lineage (Figs. 1B and 2B). The transcriptomic data suggests the presence of clones that remain after surgery and regrow with time with heterogeneous expression profiles.

### Transcription factor analysis

TF coordinates the on-and-off states of gene expression and control cell identity and cell state through core transcriptional regulatory circuitry [10]. Therefore, we performed iRegulon analysis to identify potential TF with the highest Network Enrichment Score (NES) showing regulation of the main metabolic events that were found altered in our cohort. In the metastatic corticotroph PitNET *ELK3 (NES* = *4.567)* and *HNF4A (NES* = *4.719)*, which potentially participate in metabolic gene regulation, were equally expressed in both, the primary and the recurrent lesions (p = 0.651 and p = 0.086, respectively). *PPARG*, *SREBF1*, and *STAT1* were similarly expressed in both, the primary and the recurrent silent corticotroph PitNET (NES 4.283, 6.489, and 4.294, respectively (*p* = *0.89, p* = *0.138, p* = *0.133,* respectively). The somatotroph PitNET expressed the TF’s *XBP1 (NES* = *5.499), ESRRA (NES* = *5.025)* and *ZBTB33 (NES* = *4.329)*, which potentially regulate important metabolic genes (Fig. 3G) and no differential expression between the primary and recurrent lesions was observed (p = 0.090, p = 0.315 and p = 0.357, respectively). The TFs found to be relevant to the biology of gonadotroph PitNET were *PPARG (NES* = *5.399), ZBTB3 (NES* = *5.234)* and *SREBF1 (NES* = *9.452),* from which only *PPARG* was up-regulated in primary tumors (p = 0.004, p = 0.733 and p = 0.271, respectively) (Fig. 3C).

### scRNAseq show different cell populations in PitNET

**Fig. 4:**
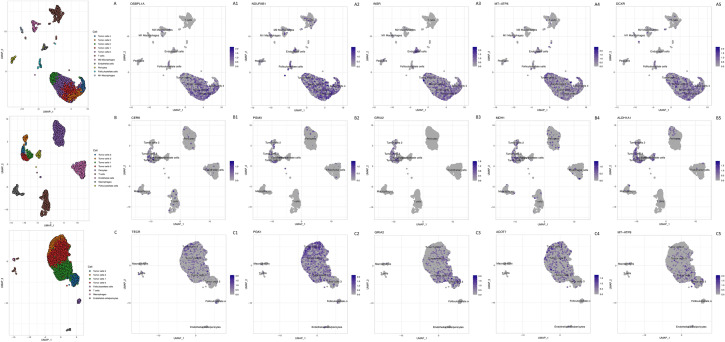
Panel **A** cell clusters identified by scRNAseq in two corticotroph PitNET, showing macrophages, pericytes, endothelium, folliculostellate cells and tumor cells. Panels **A1**–**A5** expression of metabolism-related genes: *OSBPL1A, NDUFAB1, INSR, MT-ATP8 and DXCR*, respectively, in corticotroph PitNET cells. Panel **B** cell clusters identified by scRNAseq in somatotroph PitNET, showing macrophages, pericytes, endothelium, folliculostellate cells and tumor cells. Panels **B1**–**B5** expression of metabolism-related genes: *CERK, PGM3, GRIA2, MDH1 and ALDH1A1*, respectively. Panels **C1**–**C5** expression of metabolism related genes: *TECR, PGK1, GRIA2, ACOT7 and MT-ATP8* in nonfunctioning gonadotroph PitNET

Since bulk transcriptomics analyzes cell populations comprising different clones, we conducted scRNAseq analysis of publicly available data, looking specifically for the transcriptomic signature of individual tumoral cells. We analyzed one gonadotroph, one somatotroph and two corticotroph PitNET, all of them measuring 1 cm or less. This single cell transcriptomic analysis allowed us to identify the presence of macrophages (CD163), T cells (CD3D), and pericytes (PDGFRB), as well as endothelial (VWF) and folliculostellate (SOX2) cells, within the tumor microenvironment. We also corroborated the expression of the different canonical hormones and TF, specific for each pituitary lineage: TBX19 (T-PIT) and POMC in corticotroph PitNET, POU1F1 (PIT-1) and GH in somatotroph PitNET, and NR5A1 (SF1), LH and CGA in gonadotroph PitNET (Additional file 2: Fig. S2). Interestingly, somatotroph and gonadotroph PitNET showed four different cell populations (clusters 0–3) (Fig. 4B and C, respectively), whereas the corticotroph PitNET showed five cell clusters (cluster 0–4) comprising the entire tumor mass (Fig. 4A). The corticotroph PitNET cells showed expression of OSBPL1A which is involved in lipid transport, NDUFAB1 and MT-ATP8 which participate in mitochondrial metabolism, and DCXR which participates in L-xylulose reductase reactions (Figs. 4A1, 5). The four cell clusters from the somatotroph PitNET showed the expression of genes involved in ceramide metabolism (*CERK*), *PGM3* which encodes phosphoglucomutase 3, a key enzyme in the glycosylation pathway, and MDH1 which encodes malate dehydrogenase, an enzyme that converts malate to oxalate (Figs. 4B1, 5). The genes found to be expressed among the four gonadotroph PitNET clusters included *ACOT7* (encoding acyl coenzyme A thioester hydrolase) and *TECR* (encoding trans-2,3-enoyl-CoA reductase), which participate in long chain fatty acid metabolism; *MT-ATP8* (encoding ATP synthase F0, subunit 8) responsible for the final step of mitochondrial oxidative phosphorylation and electron transport, and *GRIA2* (glutamate receptor 2) which is essential in glutamate transport (Figs. 4C1, 5).

**Fig. 5:**
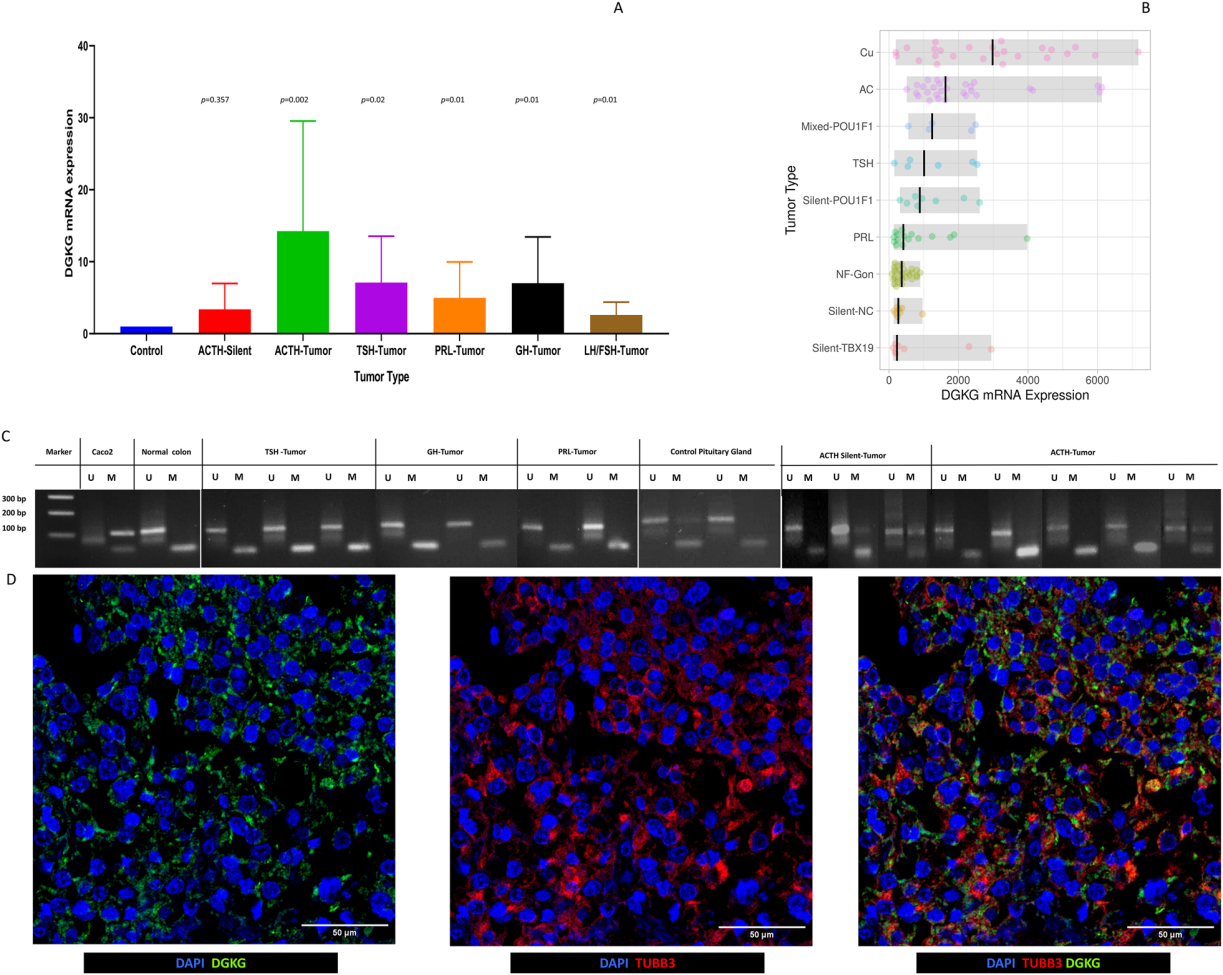
Panel **A** RT-qPCR validation of *DGKG* gene expression in corticotroph, somatotroph, lactotroph, thyrotroph, and gonadotroph PitNET from a second independent cohort. Panel **B** *DGKG* gene expression in a third independent cohort from E-MTAB-7768, confirming our transcriptome and RT-qPCR findings of *DGKG* gene expression. Panel **C** methylation specific PCR from the second cohort showing no differences in DNA methylation patterns, correlating with methylome analysis from recurrent tumors. Panel **D** immunofluorescence results from DGKG protein in somatotroph PitNET

All tumors showed cells that expressed metabolism-related genes, and several cell clusters per tumor.

### Genomic stability between primary and recurrent tumors

We assessed the potential genomic evolution through time in primary and recurrent tumors from the same patient and looked for mutations in the transcriptomically altered genes by means of whole exome sequencing. The paired primary and recurrent tumors were sequenced at 100X depth, and approximately 95% of the reads were correctly mapped. We identified approximately 10,600 SNV in each of the tumors analyzed, be it primary or recurrent (Additional file 3: Fig. S3). We observed less than 5% of genomic changes in the SNV profile in the recurrent tumor compared to their respective primary lesion (Figs. 1 and 2).

We searched for genes known to have SNV in other cohorts and other PitNET subtypes. All of the gonadotroph PitNET showed in both, the primary and the recurrent lesions, SNV in the *AIP* (Aryl hydrocarbon receptor interacting protein rs64108, c.C682A, p.Q228K) and *MEN1* (Multiple endocrine neoplasia type 1 rs2959656, c.A1621G, p.T541A) genes (Fig. 1D–F). No other known genomic variant related to sporadic PitNET was found among gonadotroph PitNET (Additional file 1: Fig. S1). In only one pair of tumors, extensive genomic differences in the recurrent tumor that were not present in the primary tumor, were found such as *CDKN1B* (rs146973564, c.C365T, p.P122L) and *MLH1* (rs1799977, c.A655G, p.I219V) among others (Fig. 1D–F). Interestingly, two of the patients who had received radiotherapy between the primary and recurrent tumor surgeries showed little genomic changes in their exomic profiles.

The exomic abnormalities found in the metastatic corticotroph PitNET included two *USP8* variants (rs672601311, c.C2159G, p.P720R, and rs11638390, c.A2215G, p.T739A), an *ATRX* variant rs3088074 (c.G2785C, p.E929Q) and a deletion (exon15:c.4377_4379del:p.E1464del), as well as SNV in *TP53* (rs1042522, c.C215G, p.P72R), and *EGFR* (rs2227983, c.G1562A, p.R521K) (Fig. 2D–F) in both, the primary and recurrent tumor samples, along with the SNV profile we have previously described in the recurrent metastatic corticotroph PitNET [11], with negligible differences between the primary and the recurrent lesions, despite a MKi67 of 80% in the recurrent neoplasm, and the fact that the patient was treated with radiotherapy and temozolomide. Both, the primary and recurrent silent corticotroph PitNET showed similar SNV in genes such as *USP8* (rs11638390, c.A2215G, p.T739A), *ATRX* (rs3088074, c.G2785C, p.E929Q), and *MSH6* (rs1042821, c.G116A, p.G39E) (Fig. 2D–F). The somatotroph PitNET showed a high degree of genetic heterogeneity between the primary and the recurrent tumors with extensive genomic differences in more than 40% of the SNV profiles; SNV were found in *AIP* (rs641081, c.C682A, p.Q228K), in both, the primary and the recurrent lesion (Fig. 2D–F), whereas the SNV found in *GNAS* (rs11554273, c.C601T, p.R201C) and *GPR101* (rs1190736, c.G370T, p.V124L) were present only in the recurrent tumor.

The primary and recurrent tumors were genomically similar to each other, but not to other neoplasms (Figs. 1, 2 and Additional file 1: Fig. S1), showing a stable genomic landscape and little modifications through time.

### Methylation analysis

DNA methylation defines cell state and lineage by controlling gene expression [12]. Therefore, after establishing the epigenetic profiles of lincRNA, we sought for other potential epigenetic regulatory mechanisms such as DNA methylation.

All the analyzed DNA methylation events were lower than the established threshold, and non-significant methylation events were documented in most of the tumor pairs. Only the somatotroph and the gonadotroph PitNET (which already has different exomic profiles) showed extensive DNA methylation differences when comparing primary and recurrent lesions (Figs. 1C and 2C).

A reduced number of differentially methylated genes were found among the primary and recurrent lesions from the non-functioning gonadotroph PitNET, the metastatic corticotroph PitNET, and the silent corticotroph PitNET, independently of cellular lineage (Additional file 1: Fig. S1). Most primary and recurrent tumors from the same patient clustered together, showing similar methylation profiles, which is illustrated in Fig. 1C that depicts the gonadotroph PitNET. The primary and recurrent silent corticotroph PitNET and the metastatic corticotroph PitNET also clustered together, while the primary and recurrent lesions from the patients with the somatotroph PitNET displayed a widely different methylation profile (Fig. 2C). We then looked for the metabolism-related genes altered in the transcriptome analysis to determine if they are potentially regulated by methylation, and again, we did not find any of the aforementioned genes with differential methylation profiles.

### DGKG up-regulation in two other independent cohorts

*DGKG* was up-regulated in the recurrent somatotroph and the silent corticotroph PitNET recurrent tumors as well as in the primary metastatic corticotroph PitNET, but not in the gonadotroph PitNET. According to our current enrichment analysis, DGKG participates in at least three metabolic pathways, phosphatidylinositol signaling, phospholipase D signaling as well as in glycerophospholipid metabolism which we previously described altered in corticotroph and somatotroph PitNET [13]. Therefore, we evaluated a second, previously described cohort of unrelated PitNET, including corticotroph, lactotroph, thyrotroph, somatotroph and gonadotroph tumors, to validate *DGKG* mRNA expression by RT-qPCR, although we did not formally test it as a relapse marker [7]. *DGKG* gene expression was upregulated in somatotroph (p = 0.01, independently of *GNAS* mutations), thyrotroph (p = 0.02) and lactotroph (p = 0.01) PitNET when compared to non-tumoral gland (Fig. 5A). In TBX19 (T-Pit)-derived PitNET, *DGKG* gene expression was upregulated in the corticotroph PitNET causing CD (*p* = 0.002), but only in one of the three silent corticotroph PitNET (*p* = 0.357) (Fig. 5A). Twelve of the 20 gonadotroph PitNET from the second cohort showed up-regulation of *DGKG* mRNA (p = 0.01). We also found it more readily up-regulated in functioning PitNET when compared to non-functioning PitNET (p = 0.0008). DGKG protein expression by immunofluorescence was also validated in all lineages of PitNET (Fig. 5D). In a third independent cohort, corresponding to publicly available data from MTAB-7768, *DGKG* gene expression was also found to be upregulated in corticotroph, thyrotroph, and somatotroph PitNET, as well as in mixed POU1F1 (PIT-1) PitNET, but not in gonadotroph, silent corticotroph and null cell PitNET (Fig. 5B).

*DGKG* gene expression is potentially regulated by methylation DNA promoter regions [8], therefore, we analyzed *DGKG* DNA promoter methylation, by methylation specific PCR. We used non-tumoral colon and colon cancer cell line CACO2 as DGKG promoter methylation controls as described previously [8]. We did not observe any differences in methylation profiles among tumors regardless of their cellular lineage, meaning that somatotroph, thyrotroph, lactotroph, and corticotroph PitNET showed no differences in promoter methylation compared to non-tumoral pituitary gland (Fig. 5C).

### DGKG as therapeutic target in PitNET: apoptosis induction and proliferation inhibition

Due to the potential role of *DGKG* in PitNET biology, we decided to perform drug-gene interaction analysis to find potential drugs that could be repurposed as alternative therapies for these neoplasms. By means of virtual screening, which comprises molecular docking and ADMET analysis, we identified the following FDA-approved drugs that could be repurposed to target *DGKG*: imatinib, nilotinib, dasatinib (Additional file 4: Fig. S4), pimozide, dihydroergotamine, paliperidone and avatrombopag. These TKI have already been found to be successful in other neoplasms. Drug concentrations were selected based on previous reports and the fact that even at low doses they can inhibit the activity of several molecules [14, 15].

**Fig. 6:**
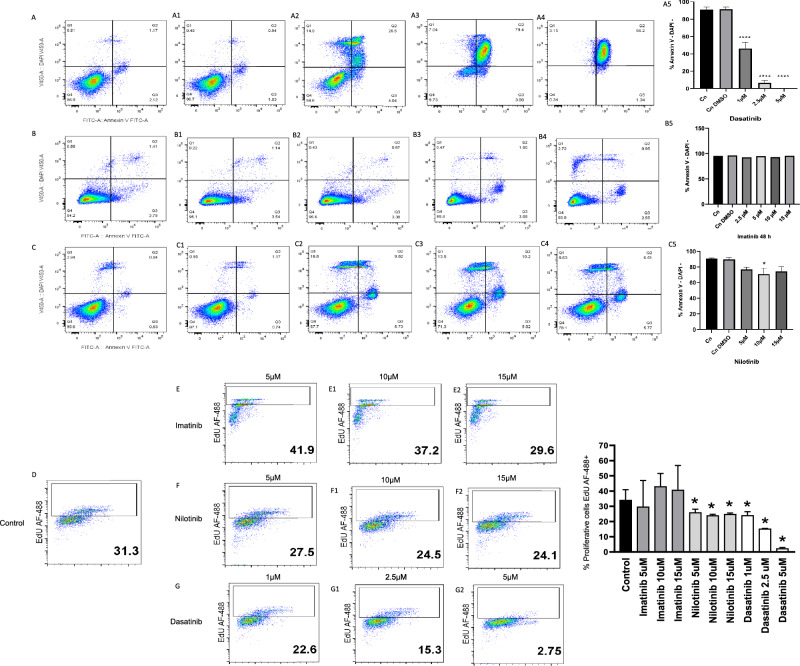
Panel **A** GH3 cells without any treatment as control (Cn), panel **A1** GH3 cells with DMSO vehicle only, both, showing no apoptosis induction; panels **A2**, **A3**, and **A4** apoptosis induction by dasatinib treatment at 1, 2.5 and 5 μM concentrations. Panel **A5** graph statistical results from dasatinib treatments. Panels **B**–**B5** and **C**–**C5** no apoptosis induction by imatinib and nilotinib, respectively. Panel **D** GH3 cell culture without any TKI treatment whereby 31.3% of live cells show proliferation; imatinib treatment did not result in any reduction in proliferation, panels **E**–**E2**. nilotinib treatment showed 12–22% reduction of cell proliferation **F**–**F2** panels. dasatinib induces a 28% and 98% reduction of cell proliferation at different concentrations, panels **G**–**G2**

Dasatinib, nilotinib and imatinib showed a strong electrostatic interaction with the active phosphorylation binding site of the DGKG protein (Additional file 4: Fig. S4). Apoptosis was induced in GH3 cells after exposure to minimum dasatinib concentrations (p = 0.0001) (Fig. 6A), whereas imatinib and nilotinib (p = 0.2501) had no effect (Fig. 6B–C). Dasatinib resulted in a significant dose-dependent increment in cell death (p = 0.0001) (Fig. 6A). Dasatinib significantly reduced cell proliferation in a dose-dependent manner by 28% at 1 μM (p = 0.0048), by 50% at 2.5 μM (p = 0.003) and by 98% at 5 μM (p = 0.0395) (Fig. 6D–G). Nilotinib induced a 12–22% reduction in cell proliferation at 5 μM (p = 0.0011), 10 μM (p = 0.0011) and 15 μM (p = 0.0011) concentrations (Fig. 6D–G). Imatinib did not have any effect on PitNET cell proliferation (p = 0.999) (Fig. 6D–G). These results indicate that dasatinib induces apoptosis and inhibits cell proliferation in PitNET at very low concentrations and may translate into a potentially safe and effective drug in these patients.

## Discussion

In the present work we longitudinally analyzed the transcriptome, exome and methylome of primary and recurrent PitNET from the same patient and validated our findings in two additional independent cohorts. Primary and recurrent tumors differed transcriptomically, whereas their genomic and methylomic profiles did not. Even though PitNET have traditionally been considered monoclonal epithelial neoplasms [16], our results suggest that these lesions are composed of diverse clones, some of which may linger on after the initial surgery and can potentially regrow subsequently. Our results are in line with a recently published single cell RNA sequencing study, that described the presence of different cell subpopulations in PitNET [17]. Currently, tumors in general are known to be heterogeneous tissues consisting of many distinct cell types in spatially complex arrangements [18]. In many biological and clinical settings such cellular heterogeneity plays a critical role not only in primary tumor oncogenesis, but also in the development of recurrences and metastasis and hence, must be taken into account when deciding treatment alternatives [19]. A growing body of evidence indicates that cancer progression at the cellular level is, in essence, an evolutionary process. During tumor progression, novel phenotypic variants emerge via heritable changes in gene expression, and subsequently, phenotypic variants are subject to natural selection under the action of tumor microenvironment [20]. Intratumoral transcriptomic heterogeneity can confer selective advantages that influence the phenotype of tumor cell populations and thus, their biological behavior [21]. These transcriptomic signatures govern crucial biological events including glucose and lipid metabolism, cellular proliferation, and apoptosis [21].

In general, recurrences are largely driven by cells that survive therapeutic interventions [22]. Our results showed transcriptomically heterogenous profiles of primary and recurrent tumors. Transcriptional differences between primary and recurrent tumors have previously been described in other types of neoplasms such as head and neck cancer [23], hepatocarcinoma [24] and breast cancer [25]. In PitNET and other neoplasms these transcriptomic profiles have shown altered metabolic events and are related to recurrence. In the case of PitNET, tissues showing cell clusters with altered lipid metabolism show higher recurrence rates [17, 25]. Gene expression changes reveal altered lipid metabolism as a hallmark of the cells that survive tumor regression [25]. Most of the altered pathways in recurrent PitNET are related to metabolism of several molecules, including lipids, purine, pyrimidine, and carbohydrates. Lipids are used as energetic sources to fuel tricarboxylic acid cycle, as structural molecules making up cell membranes, and as intra- or extracellular signaling molecules [26]. High levels of purine and pyrimidine metabolism promote tumorigenic capacity and contribute to recurrence [27, 28]. Purines can be used to synthetize DNA, RNA, and as cofactors in crucial biochemical reactions, and they also have a role in energy generation [29]. Reprogramming of carbohydrate metabolism, particularly glucose, provides energy and important substrates for cell proliferation, metastasis, and immune evasion of tumor cells. It also provides cells with intermediate molecules required for biosynthetic pathways including ribose for nucleotide synthesis, and glycerol and citrate for lipid synthesis [30].

The gene regulatory network analysis showed several TF that could regulate metabolism-related gene expression in PitNET. Dysregulated TF mediate aberrant gene expression [31]. Hepatic nuclear factor 4 alpha (HNF4A) is a TF that could participate in PitNET metabolism regulation*,* as it is known to participate in glycolysis, gluconeogenesis, fatty acid metabolism and apolipoprotein synthesis among other metabolic pathways while it is also related to cellular proliferation, differentiation, and tumor progression. Abnormalities in lipid metabolism and small molecule biochemistry have previously been reported in PitNET [32], as well as in several other neoplasms such as gastric, colorectal, liver, pancreatic and lung cancer [33]. *ELK3* participates in the regulation of mitochondrial metabolism regulation [34], and its expression has been found to be associated with a poor prognosis in patients with gliomas [35]. *PPARG, SREBF1* and *STAT1* are involved in energy balance, cholesterol, fatty acid, triacylglycerol, and phospholipid biosynthesis as well as in glycolysis, citrate cycle and oxidative phosphorylation [36–38]. These TF are expressed in PitNET and they regulate metabolic traits related to aggressiveness and recurrence [17, 39]; they also are expressed and may play important pathogenic roles in various tumor types [38, 40, 41]. *ESRRA* and *XBP1* are known to regulate glucose, lipid, and mitochondrial metabolism [42, 43] while *ZBTB33* is related to cell cycle progression [44] and they have been found to be expressed in several types of cancer [43–45].

LincRNA are transcripts longer than 200 nucleotides that undergo alternative splicing producing numerous non-coding isoforms, which are transcribed by RNA-pol III, have a 5’ end cap, and a 3’ poly-A tail, as well as the ability to exert regulatory functions through elaborate structures [46]. The lincRNA’s can influence several aspects of tumor biology such as metabolism, proliferation, apoptosis and invasion [46]. Several lincRNA’s have been shown to be altered in PitNET and have a distinctive expression pattern according to the TF driving pituitary cell differentiation [7]. Some lincRNA’s could predict tumor recurrence [47]. *LINC01619* has been described to be up regulated in non-small cell lung cancer enhancing cell viability, cloning ability and stemness, which is characterized by an increased number of ALDH + cells [48]. *LINC00342* has been found to be upregulated in colon adenocarcinoma promoting cell proliferation and invasion [49], whereas *LINC02691* and *LINC00486* expression correlates with overall survival and prognosis in hepatocellular and gastric carcinoma, respectively [50, 51].

The finding of an upregulated *DGKG* gene expression, merits special consideration. *DGKG* codes for an enzyme that catalyzes the phosphorylation of DAG (diacylglycerol), an essential lipid second messenger into phosphatidic acid (PA) [52]. It can directly interact with ABL to modulate its function, and through the conversion and regulation of DAG and PA, DGKG could mediate signaling through PKC, PKD, RAS,GRP1, MUNC13, chimarins, mTOR, RAF1, PIKFYVE, PP1, PTPN6, ABI1, SLC31A1, AKT1, MAPK1, HIF1A, MYC, SREBP, SPHK1-2 and influence proliferation, apoptosis, anergy in T cells, cytoskeleton rearrangement, glucose and lipid metabolism and granule maturation and secretion [53–55]. Members of diacylglycerol kinase family, as well as PA can activate SRC, promoting cell proliferation [54]. Through the regulation of DAG and PA, DGKG could influence signaling pathways such as RHOA, NFKB, MAPK1, mTOR and MAPK1 through several of the previously mentioned molecules [52–55].

Dasatinib can inhibit some of the molecules and pathways in which DGKG potentially participates directly or indirectly such as SRC, AKT1 and ABL1, and can also regulate other genes expressed in aggressive tumors such as LCK, YES1, ABL1, KIT, PDGFRB, PTK2 and EPHA2 [14, 56], some of which we found expressed in PitNET. Dasatinib can induce apoptosis and reduce cellular proliferation through several pathways and is capable of inhibiting GH secretion [57–59]. Thus, our results suggest that this TKI, used at low doses to minimize side effects, may represent a promising alternative therapy for aggressive PitNET and perhaps, pituitary carcinomas.

The exome profiles of both corticotroph PitNET showed an ATRX gene variant that has been previously related to tumor susceptibility [60] although, it has not been related to corticotroph PitNET previously [61]. Interestingly, the USP8 variant found in the metastatic corticotroph PitNET is related to increased risk of recurrence [62]. Whereas the somatotroph PitNET showed an allelic variant in *AIP* which only one study has related to sporadic and hereditary somatotropinomas [63], but it was not present in other larger familial isolated pituitary adenoma studies [64]. Further studies are needed to elucidate the biological meaning of these allelic variants found in our cohort.

We acknowledge the limitations of our study due to the reduced sample size, and the fact that we did not perform tumor differentiation analysis. However, it is important to realize that having available for molecular studies primary and recurrent/persistent tumors from the same patient is an infrequent scenario. Thus, despite the limited number of patients, our results are in line with well-established molecular mechanisms and the biological implications of our findings are also relevant for the future design of novel therapies.

## Conclusions

Our study shows that PitNET are genomically and methylomically stable through time, indicating that mechanisms other than somatic mutations are involved in pituitary tumorigenesis and that their biology could be driven by transcriptomically heterogeneous clones within the tumor itself. Dasatinib represents an attractive pharmacological therapy for aggressive PitNET.

### Supplementary Information

**Additional file 1**: **Fig. S1**. Panel **A** transcriptome, **B** methylome and **C** exome of all the tissues analyzed depicting TF lineage segregation and methylome as well as exome stability and transcriptome instability through time.

**Additional file 2**: **Fig. S2**. Panel **A B** and **C** molecular markers for macrophages (CD163), T cells (CD3D) pericytes (PDGFRB), endothelium (VWF), folliculostellate (SOX2) cells and tumor cells with lineage markers TBX19 (t-Pit) for corticotroph PitNET, POU1F1 (PIT-1) for somatotroph and lactotroph PitNET, and NR5A1 (SF1) for gonadotroph PitNET.

**Additional file 3**: **Fig. S3**. MAFtools results showing summary of variants found in PitNET.

**Additional file 4**: **Fig. S4**. Dasatinib molecular docking with DGKG. Panel **A**, **B** and **C** molecular docking results from DGKG interaction with dasatinib, imatinib and nilotinib, respectively.

## Data Availability

The datasets generated and analyzed during the current study are not publicly available due to the sensitive nature of the clinical data, genomic, methylomic and transcriptomic information that could lead to patient identification and was requested by our local ethical committee to be deposited in an institutional repository and are available from the corresponding author upon reasonable request.

## Abbreviation

ABI1: Abl Interactor 1
ABL1: ABL Proto-Oncogene 1, Non-Receptor Tyrosine Kinase
ACACA: Acetyl-CoA Carboxylase Alpha
ACOT7: Acyl-CoA Thioesterase 7
ACSS3: Acyl-CoA Synthetase Short Chain Family Member 3
ADH5: Alcohol Dehydrogenase 5 (Class III), Chi Polypeptide
ADMET: Absorption, distribution, metabolism, excretion and toxicity
AIP: Aryl Hydrocarbon Receptor Interacting Protein
AKT1: AKT Serine/Threonine Kinase 1
ALDH3B2: Aldehyde Dehydrogenase 3 Family Member B2
ATP1A3: ATPase Na + /K + Transporting Subunit Alpha 3
ATRX: ATRX Chromatin Remodeler
BBB: Blood Brain Barrier
BCAT1: Branched Chain Amino Acid Transaminase 1
CAMK1D: Calcium/Calmodulin Dependent Protein Kinase ID
CD: Cushing Disease
CD163: CD163 Molecule
CD3D: CD3 Delta Subunit Of T-Cell Receptor Complex
CDKN1B: Cyclin Dependent Kinase Inhibitor 1B
CERK: Ceramide Kinase
CGA: Glycoprotein Hormones, Alpha Polypeptide
SLC31A1: Solute Carrier Family 31 Member 1
DAG: Diacylglycerol
DAPI: 4',6-Diamidino-2-phenylindole
DCXR: Dicarbonyl And L-Xylulose Reductase
DGKG: Diacylglycerol Kinase Gamma
DGUOK: Deoxyguanosine Kinase
DMEM: Dulbecco's Modified Eagle Medium
DPYD: Dihydropyrimidine Dehydrogenase
EDEM3: ER Degradation Enhancing Alpha-Mannosidase Like Protein 3
EGFR: Epidermal Growth Factor Receptor
ELK3: ETS Transcription Factor ELK3
ELOVL7: ELOVL Fatty Acid Elongase 7
EPHA2: EPH Receptor A2
MAPK1: Mitogen-Activated Protein Kinase 1 2
ESR1: Estrogen Receptor 1
ESRRA: Estrogen Related Receptor Alpha
EXO1: Exonuclease 1
PTK2: Protein Tyrosine Kinase 2
FBS: Fetal bovine serum
GNAS: GNAS Complex Locus
GPR101: G Protein-Coupled Receptor 101
GRIA2: Glutamate Ionotropic Receptor AMPA Type Subunit 2
HIF1A: Hypoxia Inducible Factor 1 Subunit Alpha
HK2: Hexokinase 2
HNF4A: Hepatic nuclear factor 4 alpha
INPP5A: Inositol Polyphosphate-5-Phosphatase A
MKI67: Marker Of Proliferation Ki-67
KIT: KIT Proto-Oncogene, Receptor Tyrosine Kinase
LCK: LCK Proto-Oncogene, Src Family Tyrosine Kinase
LDHC: Lactate Dehydrogenase C
LH: Luteinizing Hormone
LINC: Long non-coding RNA
MAN1A2: Mannosidase Alpha Class 1A Member 2
MDH1: Malate Dehydrogenase 1
MEN1: Menin 1
MLH1: MutL Homolog 1
MSH6: MutS Homolog 6
MT-ATP8: Mitochondrially Encoded ATP Synthase Membrane Subunit 8
mTOR: Mechanistic Target Of Rapamycin Kinase
MYC: MYC Proto-Oncogene, BHLH Transcription Factor
NDUFAB1: NADH:Ubiquinone Oxidoreductase Subunit AB1
NES: Network Enrichment Score
NFKB: Nuclear factor kappa B subunit 1
NR5A1: Nuclear Receptor Subfamily 5 Group A Member 1
OSBPL1A: Oxysterol Binding Protein Like 1A
PA: Phosphatidic acid
PCK1: Phosphoenolpyruvate Carboxykinase 1
PDE1C: Phosphodiesterase 1C
PDGFRB: Platelet Derived Growth Factor Receptor Beta
PGM3: Phosphoglucomutase 3
PIKFYVE: Phosphoinositide Kinase, FYVE-Type Zinc Finger Containing
PitNET: Pituitary neuroendocrine tumor
PLIP: Protein–Ligand Interaction Profiler
POU1F1: POU Class 1 Homeobox 1
PP1: Serine/threonine specific protein phosphatase PP1 catalytic subunit
PPARG: Peroxisome Proliferator Activated Receptor Gamma
RAF1: Raf-1 Proto-Oncogene, Serine/Threonine Kinase
RHOA: Ras Homolog Family Member A
RPMI: Roswell Park Memorial Institute medium
PTPN6: Protein Tyrosine Phosphatase Non-Receptor Type 6
SNV: Single nucleotide variant
SOX2: SRY-Box Transcription Factor 2
SPHK1-2: Sphingosine Kinase 1–2
SREBF1: Sterol Regulatory Element Binding Transcription Factor 1
STAT1: Signal Transducer And Activator Of Transcription 1
TBX19: T-Box Transcription Factor 19
TECR: Trans-2,3-Enoyl-CoA Reductase
TF: Transferrin
TKI: Tyrosine kinase inhibitors
TP53: Tumor Protein P53
USP8: Ubiquitin Specific Peptidase 8
VWF: Von Willebrand Factor
XBP1: X-Box Binding Protein 1
YES1: YES Proto-Oncogene 1, Src Family Tyrosine Kinase
ZBTB3: Zinc Finger And BTB Domain Containing 3
