# Supplementary figures and images for: Longitudinal multiomics analysis of aggressive pituitary neuroendocrine tumors: comparing primary and recurrent tumors from the same patient, reveals genomic stability and heterogeneous transcriptomic profiles with alterations in metabolic pathways
Source: Acta Neuropathol Commun. 2024 Aug 31;12:142. doi: 10.1186/s40478-024-01796-x (PMC11365143; doi:10.1186/s40478-024-01796-x)

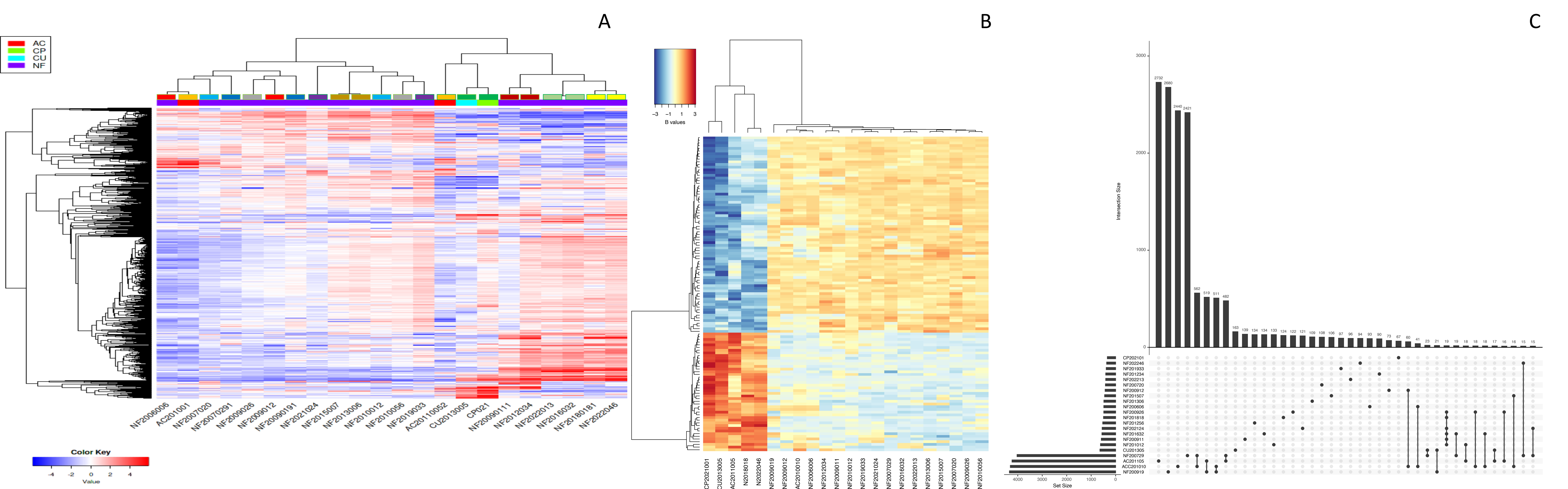

Supplement: Supplementary file 1 — Additional file 1: Fig. S1. Panel A transcriptome, B methylome and C exome of all the tissues analyzed depicting TF lineage segregation and methylome as well as exome stability and transcriptome instability through time. [file 40478_2024_1796_MOESM1_ESM.pdf]

**A**

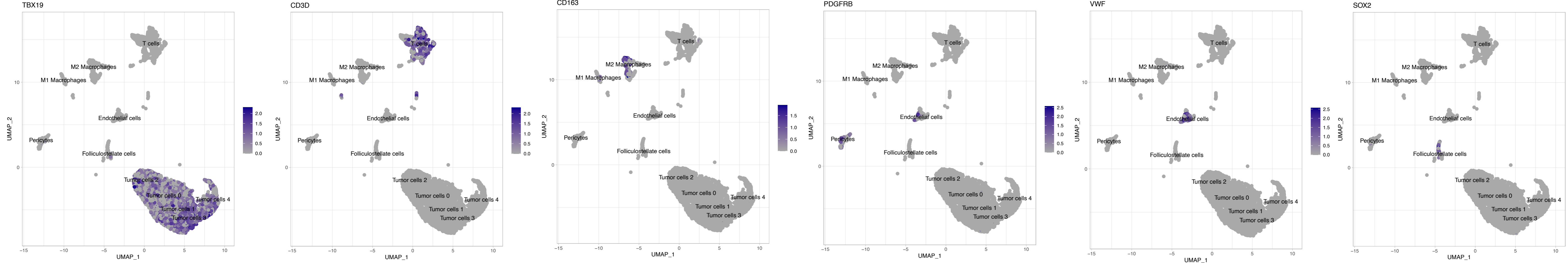

# B

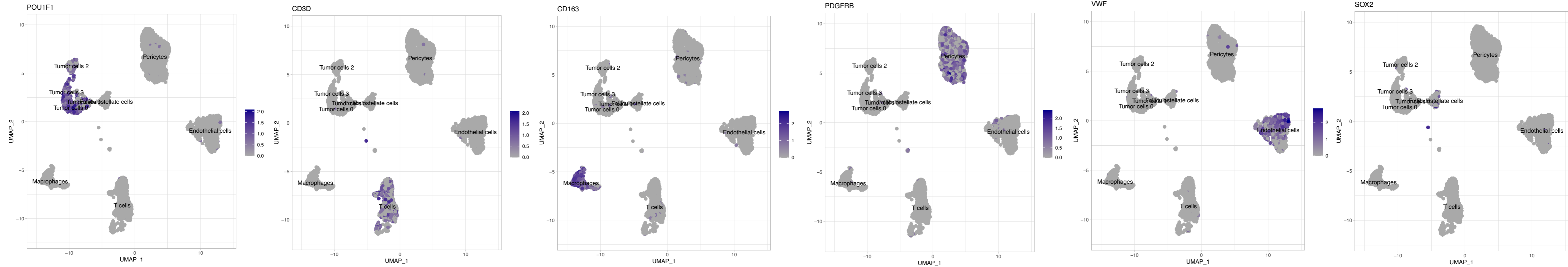

C

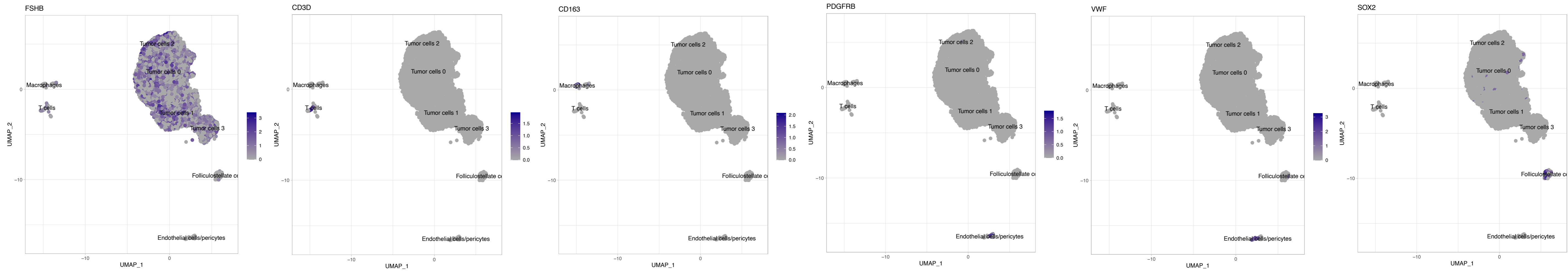

Supplement: Supplementary file 2 — Additional file 2: Fig. S2. Panel A B and C molecular markers for macrophages (CD163), T cells (CD3D) pericytes (PDGFRB), endothelium (VWF), folliculostellate (SOX2) cells and tumor cells with lineage markers TBX19 (t-Pit) for corticotroph PitNET, POU1F1 (PIT-1) for somatotroph and lactotroph PitNET, and NR5A1 (SF1) for gonadotroph PitNET. [file 40478_2024_1796_MOESM2_ESM.pdf]

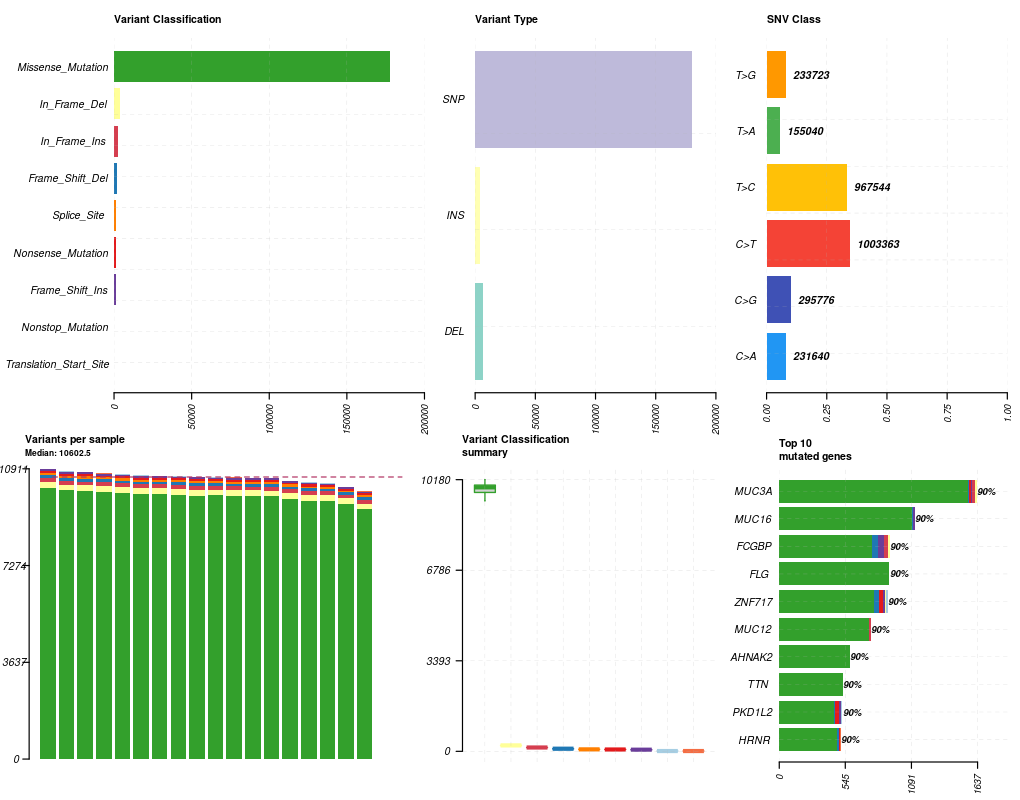

Supplement: Supplementary file 3 — Additional file 3: Fig. S3. MAFtools results showing summary of variants found in PitNET. [file 40478_2024_1796_MOESM3_ESM.png]

A

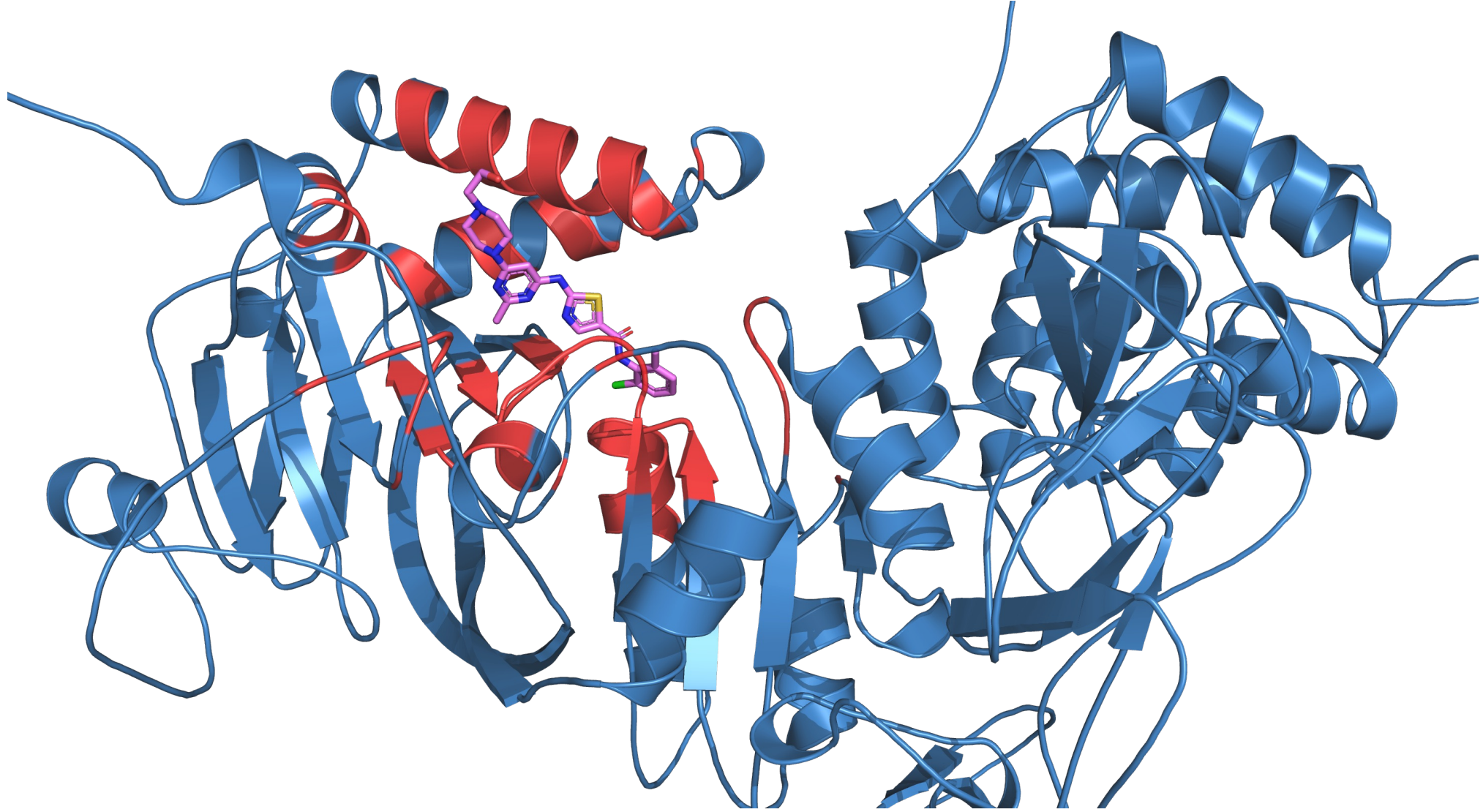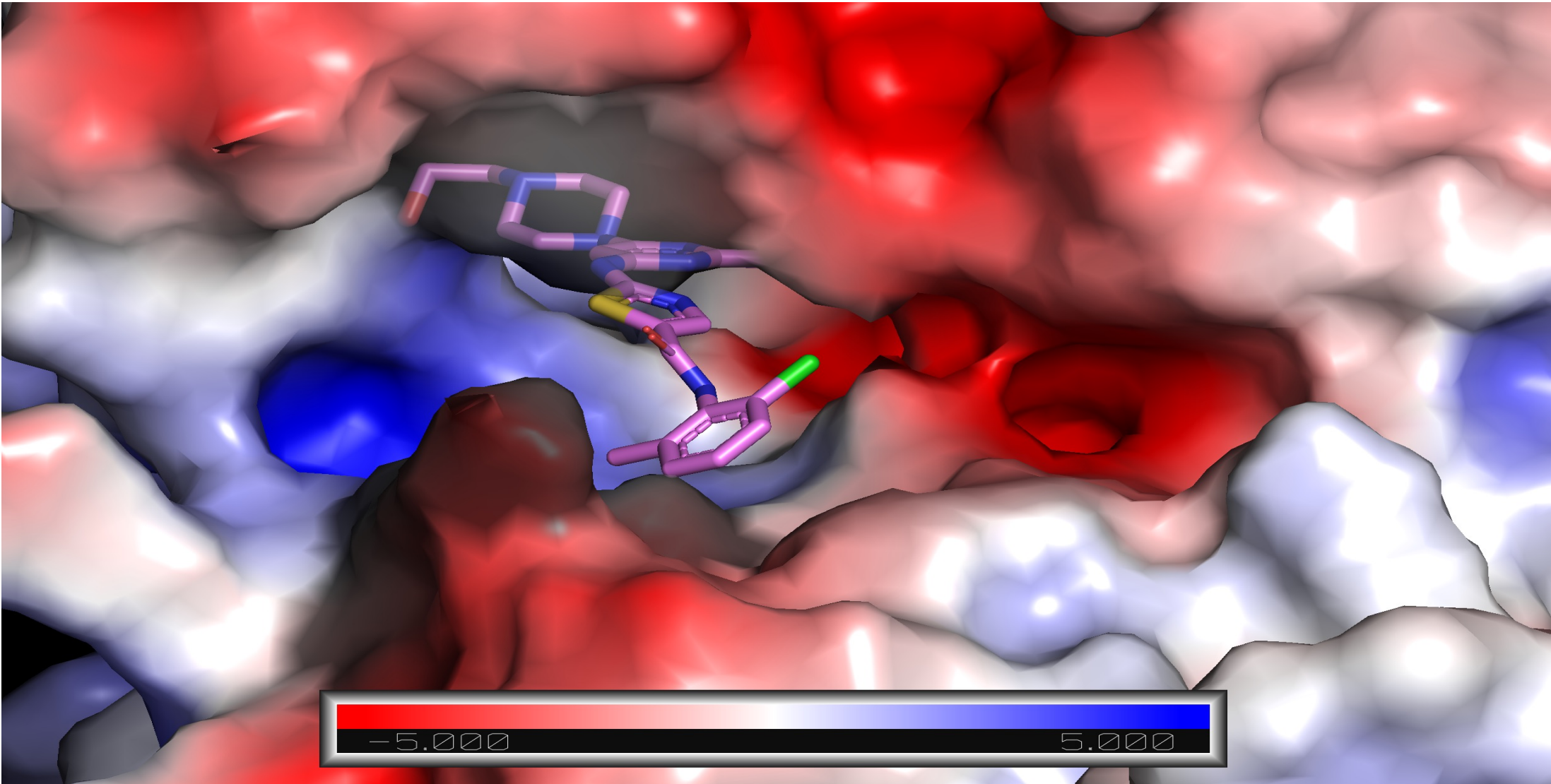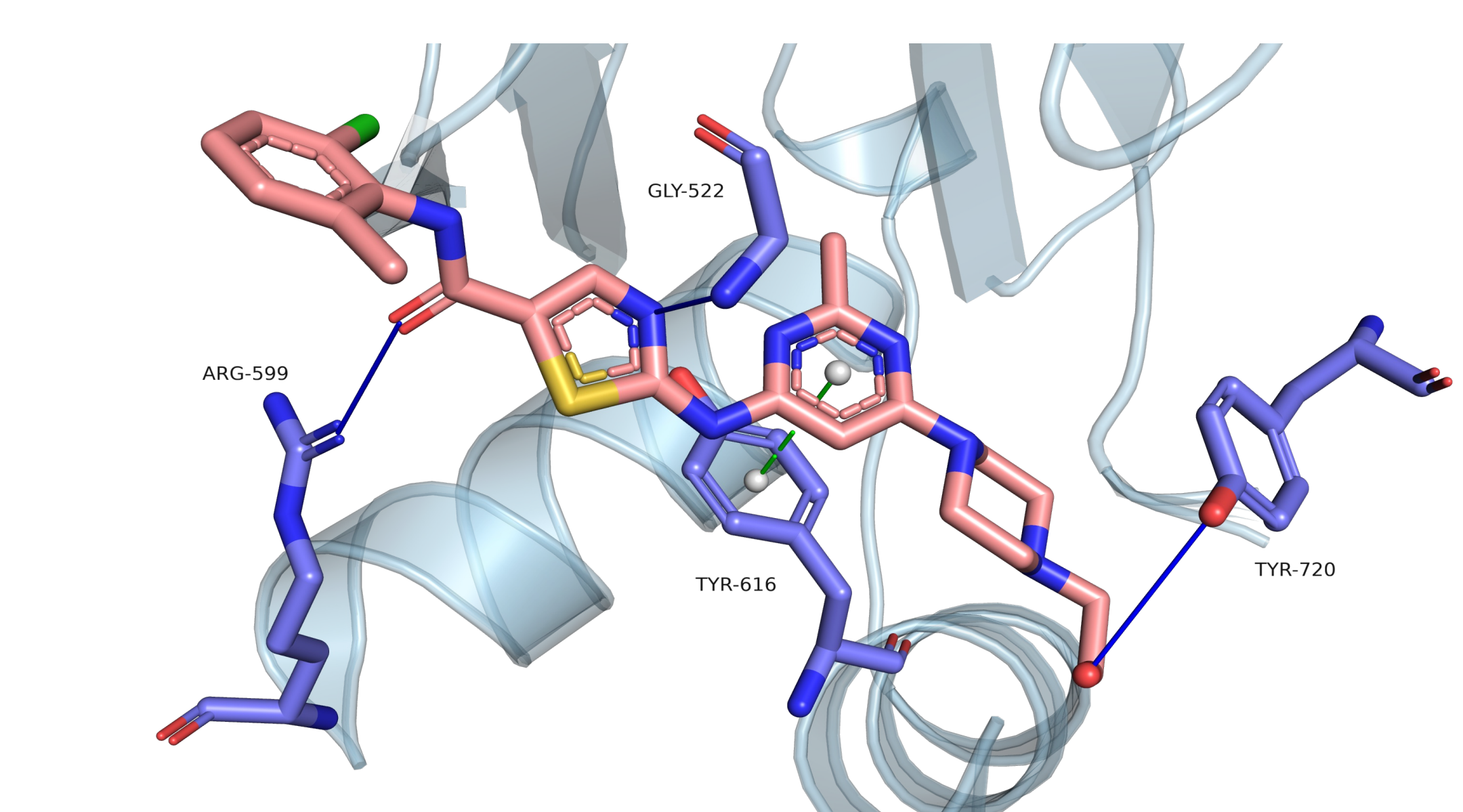

B

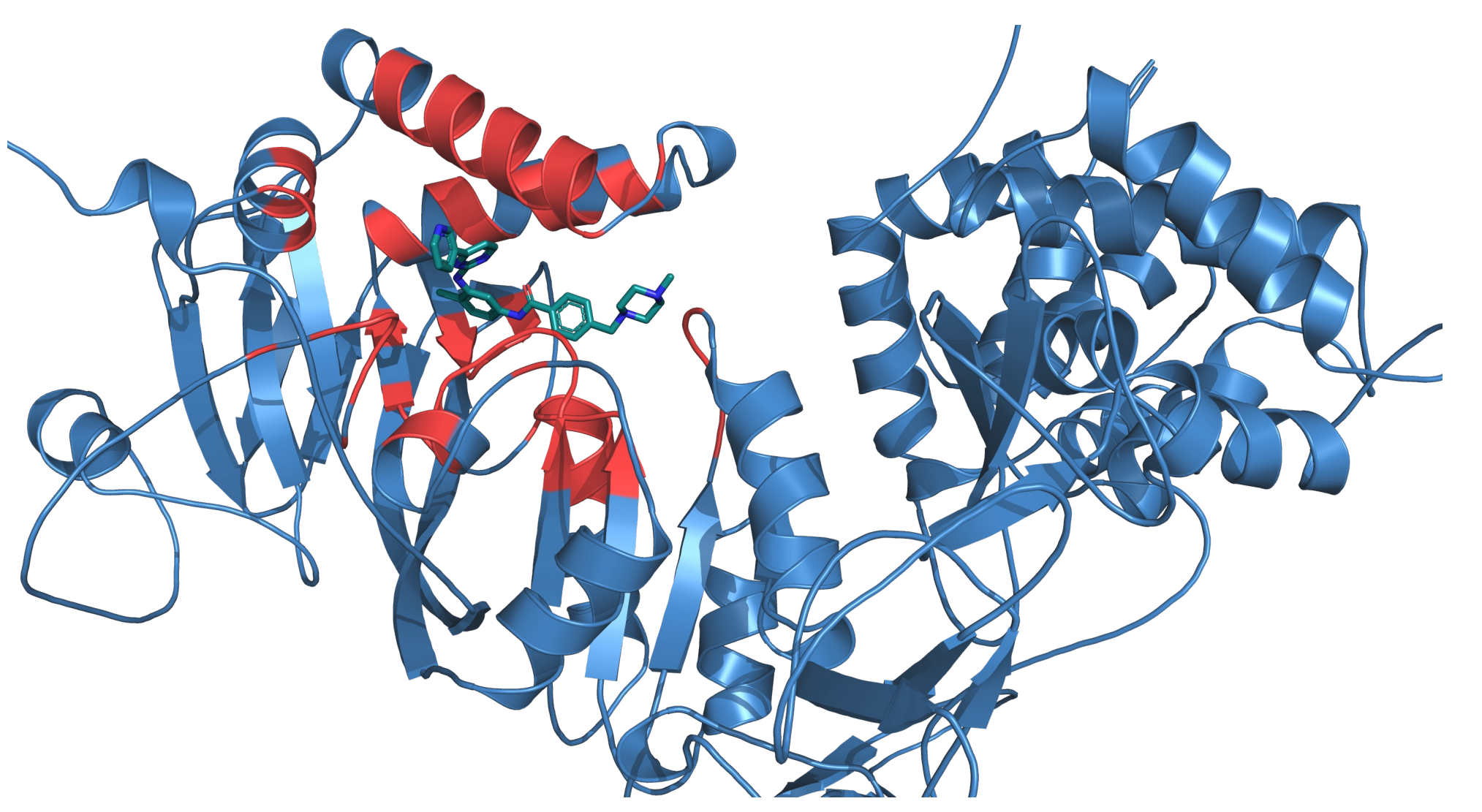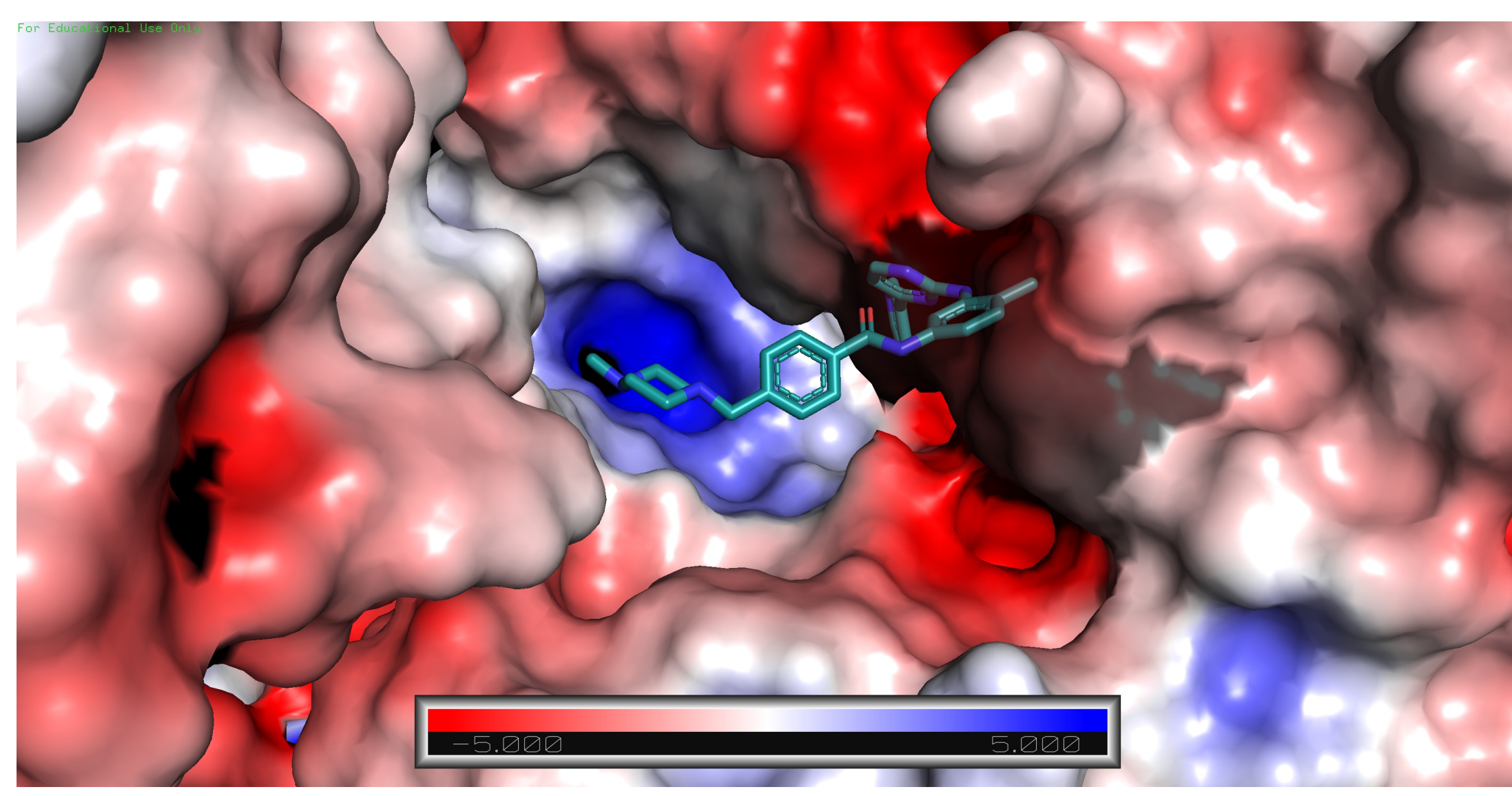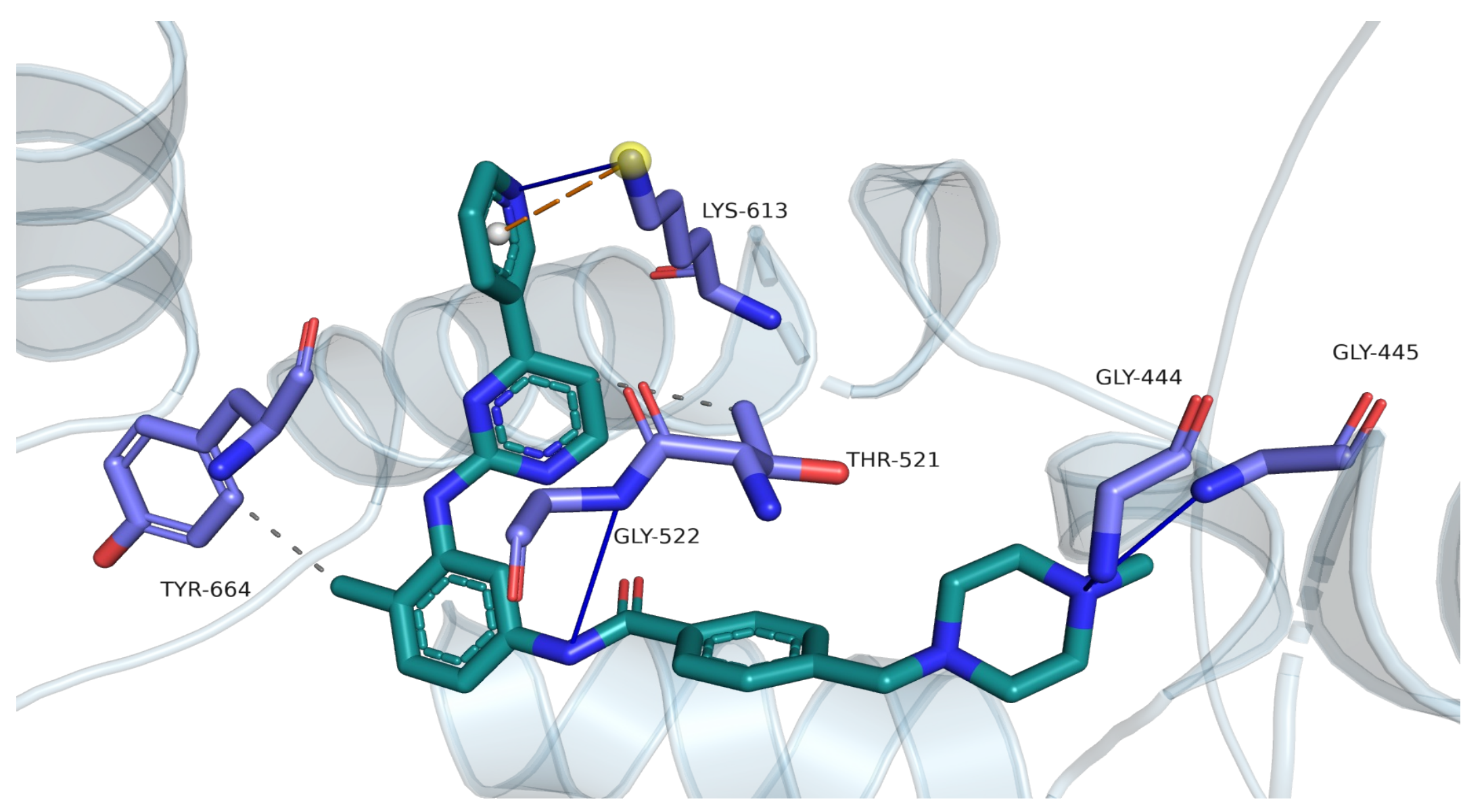

C

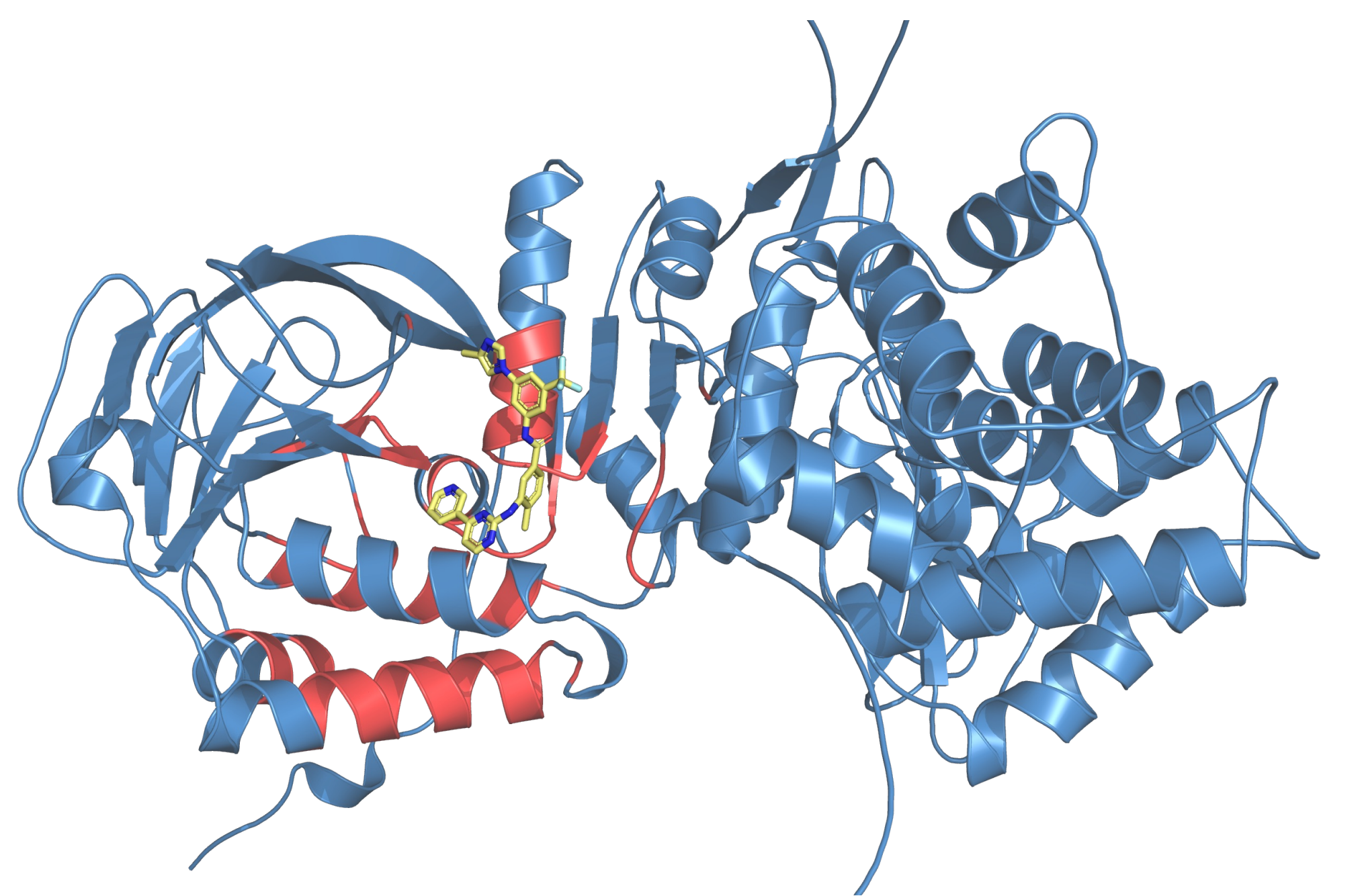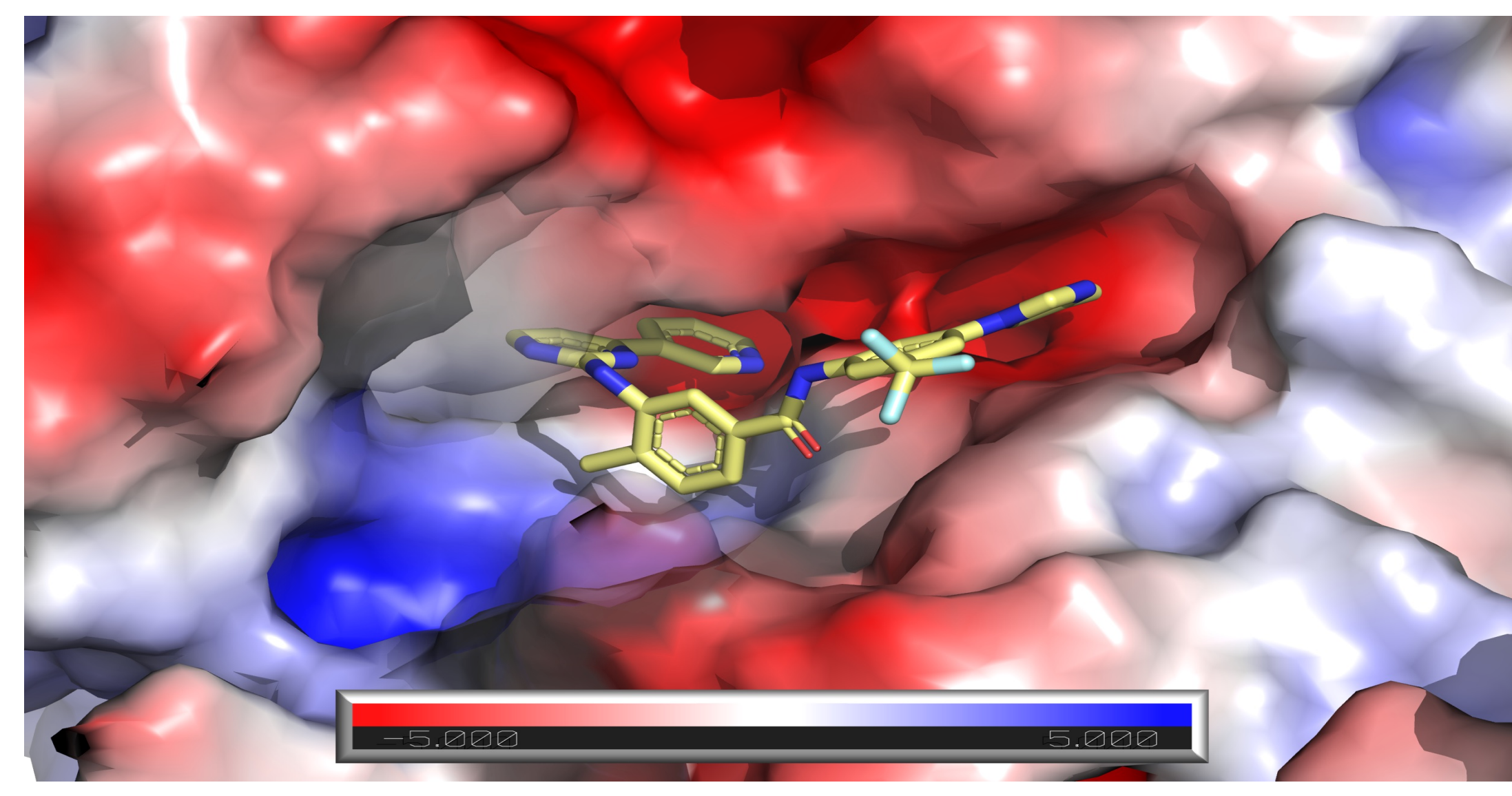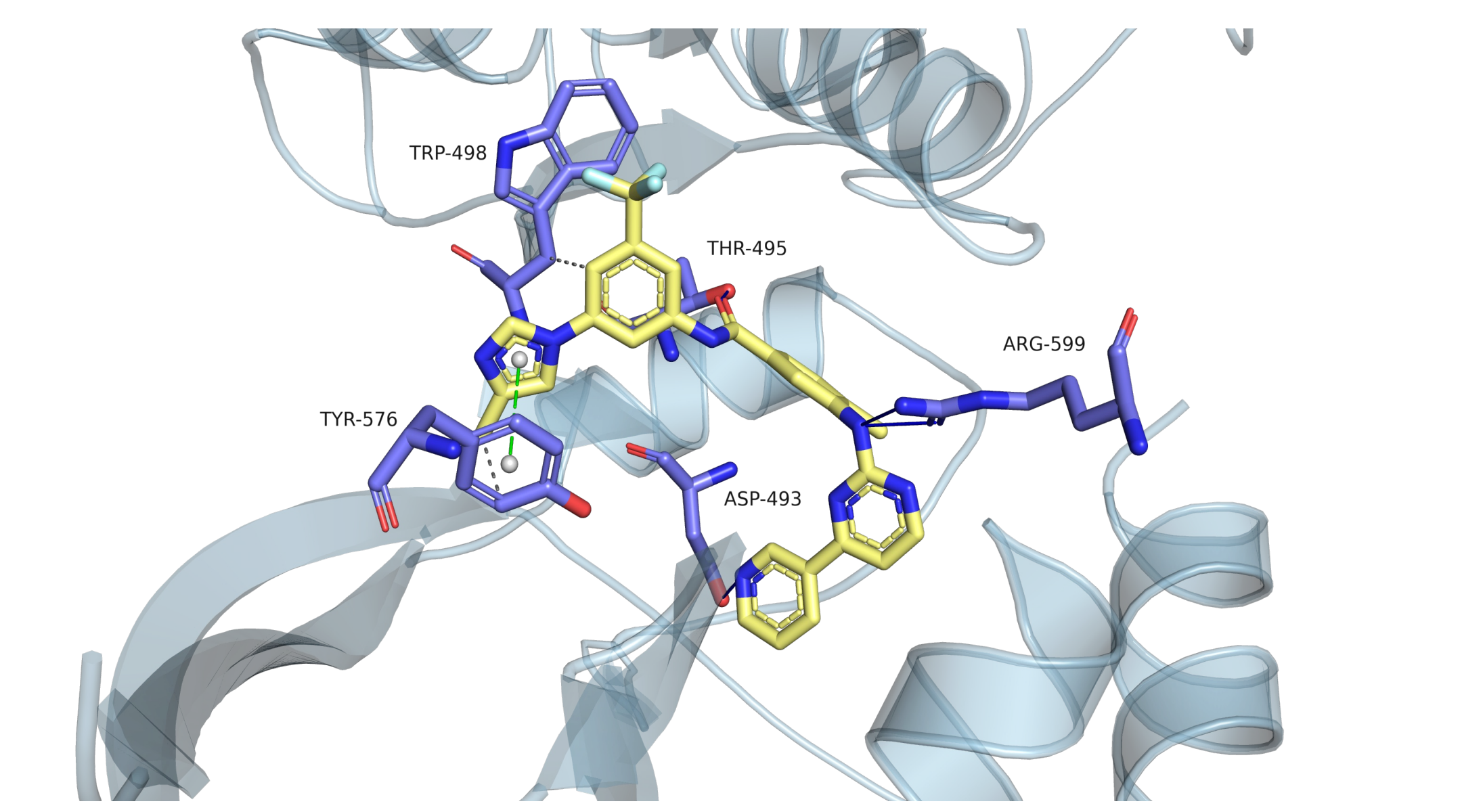

Supplement: Supplementary file 4 — Additional file 4: Fig. S4. Dasatinib molecular docking with DGKG. Panel A, B and C molecular docking results from DGKG interaction with dasatinib, imatinib and nilotinib, respectively. [file 40478_2024_1796_MOESM4_ESM.pdf]
